# Molecular phylogeny of microhylid frogs (Anura: Microhylidae) with emphasis on relationships among New World genera

**DOI:** 10.1186/1471-2148-12-241

**Published:** 2012-12-10

**Authors:** Rafael O de Sá, Jeffrey W Streicher, Relebohile Sekonyela, Mauricio C Forlani, Simon P Loader, Eli Greenbaum, Stephen Richards, Célio F B Haddad

**Affiliations:** 1 Department of Biology, University of Richmond, Richmond, VA 23173, USA; 2 Amphibian and Reptile Diversity Research Center, Department of Biology, The University of Texas at Arlington, Arlington, TX, 76010, USA; 3 Department of Environmental Sciences, University of Basel, Basel, CH-4056, Switzerland; 4 Department of Biological Sciences, University of Texas at El Paso, 500 W. University Avenue, El Paso, TX, 79968, USA; 5 Herpetology Department, South Australian Museum, North Terrace, Adelaide, 5000, South Australia; 6 Department of Terrestrial Vertebrates, Museum and Art Gallery of the Northern Territory, GPO Box 4646, Darwin, NT, 0801, Australia; 7 Departamento de Zoologia, Instituto de Biociências, Universidade Estadual Paulista (UNESP), Caixa Postal 199, Rio Claro, São Paulo, 13506-900, Brazil

**Keywords:** Microhylidae, Phylogeny, Systematics, Subfamilies, New World genera

## Abstract

**Background:**

Over the last ten years we have seen great efforts focused on revising amphibian systematics. Phylogenetic reconstructions derived from DNA sequence data have played a central role in these revisionary studies but have typically under-sampled the diverse frog family Microhylidae. Here, we present a detailed phylogenetic study focused on expanding previous hypotheses of relationships within this cosmopolitan family. Specifically, we placed an emphasis on assessing relationships among New World genera and those taxa with uncertain phylogenetic affinities (i.e., *incertae sedis*).

**Results:**

One mitochondrial and three nuclear genes (about 2.8 kb) were sequenced to assess phylogenetic relationships. We utilized an unprecedented sampling of 200 microhylid taxa representing 91% of currently recognized subfamilies and 95% of New World genera. Our analyses do not fully resolve relationships among subfamilies supporting previous studies that have suggested a rapid early diversification of this clade. We observed a close relationship between *Synapturanus* and *Otophryne* of the subfamily Otophryninae. Within the subfamily Gastrophryninae relationships between genera were well resolved.

**Conclusion:**

Otophryninae is distantly related to all other New World microhylids that were recovered as a monophyletic group, Gastrophryninae. Within Gastrophryninae, five genera were recovered as non-monophyletic; we propose taxonomic re-arrangements to render all genera monophyletic. This hypothesis of relationships and updated classification for New World microhylids may serve as a guide to better understand the evolutionary history of this group that is apparently subject to convergent morphological evolution and chromosome reduction. Based on a divergence analysis calibrated with hypotheses from previous studies and fossil data, it appears that microhylid genera inhabiting the New World originated during a period of gradual cooling from the late Oligocene to mid Miocene.

## Background

The family Microhylidae is the fourth largest anuran family (after Hylidae, Strabomantidae, and Bufonidae), consisting of 487 currently recognized species representing 8.2% of extant anuran diversity. A monographic revision of the family Microhylidae was done over 75 years ago [1]. Parker defined the family Microhylidae on the basis of 12 non-synapomorphic morphological characters and grouped the 191 species known at the time into 43 genera and 7 subfamilies: Asterophryinae, Brevicipitinae, Cophylinae, Dyscophinae, Melanobatrachinae, Microhylinae, and Sphenophryninae. Three additional subfamilies were recognized in later publications: Phrynomerinae [2], Scaphiophryninae [3], and Otophryninae [4]. A morphological review of the family analyzed 188 characters in 56 genera and 105 species [5]. All available studies show that microhylids display extensive variation in adult external morphology, osteology, and musculature at inter- and intraspecific levels. Because of this variation, phylogenetic interpretations that use morphological features have been hindered by extensive homoplasy (see review of morphological variation [6]). In many cases, the morphological convergence in microhylids is likely due to specializations associated with a burrowing lifestyle [7]. However, the monophyly of the family is supported by 20 synapomorphies derived from larval anatomy [8]. The first broad-scale attempt to examine phylogenetic relationships of the Amphibia using DNA sequence and morphology [9] used a parsimony criterion to provide support for many higher-level taxonomic rearrangements that better reflect the phylogenetic history of living amphibians and also stimulated much discussion [10]. A more recent analysis [11] expanded the sampling, both in the number of taxa and molecular markers, and using model-based analyses recovered phylogenetic relationships that were largely congruent with the earlier study [9]. Pyron and Wiens recognized 11 nominal microhylid subfamilies and several unassigned genera as *incertae sedis* within Microhylidae (mostly New World genera).

**Figure 1:**
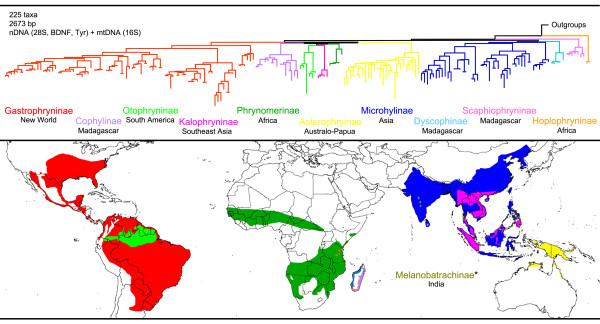
**Maximum likelihood phylogram generated from concatenated nuclear and mitochondrial DNA sequences examined for this study (top) and approximate global distribution of microhylid subfamilies (bottom) based on spatial data from IUCN et al. (2006)**. See Van Bocxlaer et al. (2006) and Trueb et al. (2011) for hypotheses related to the placement of the monotypic Melanobatrachinae (not sampled in this study).

Additionally, the following subfamilies are currently recognized [12]: Hoplophryninae and Phrynomerinae (based on [13]), Kalophryninae [14], and Otophryninae [4]. Thus, as it is currently recognized, Microhylidae is globally distributed (Figure 1) with two subfamilies occurring in the New World (Gastrophryninae and Otophryninae) and nine subfamilies occurring in the Old World (Asterophryinae, Cophylinae, Dyscophinae, Hoplophryninae, Kalophryninae, Melanobatrachinae, Microhylinae, Phrynomerinae, and Scaphiophryninae). The highest levels of diversity occur in tropical regions and three of the Old World subfamilies are endemic to Madagascar (Cophylinae, Dyscophinae, and Scaphiophryninae). Furthermore, two subfamilies possess low levels of species diversity and highly restricted geographic distributions: Hoplophryninae (two species, endemic to Eastern Arc mountains of Tanzania, Africa) and Melanobatrachinae (one species, Western Ghats of Kerala and Tamil Nadu in India).

New World microhylids (NWM) were initially included in the subfamily Microhylinae but this was demonstrated to represent a paraphyletic assemblage of both New and Old World taxa. Consequently, the subfamily Gastrophryninae was resurrected for a monophyletic clade consisting of all New World genera except *Synapturanus*[9]. Subsequent molecular analyses supported a monophyletic Gastrophryninae, though excluding *Synapturanus* and *Otophryne*[15,16]. More recently, *Synapturanus* was placed in the Otophryninae [11]. Currently, there are two subfamilies, 20 genera (nine monotypic), and 72 species of NWM [12]. To summarize, the subfamily Otophryninae includes two genera (*Otophryne* and *Synapturanus*) and five species and Gastrophryninae currently consists of 9 genera and 53 species. The two NWM genera occurring in North America were recently reviewed with examinations of phylogeographic variation: *Hypopachus*[17] and *Gastrophryne*[18]. The currently recognized species in each genus of NWM, are (with number of species in parentheses): *Adelastes* (1 sp.), *Altigius* (1sp.), *Arcovomer* (1 sp.), *Hyophryne* (1 sp.), *Melanophryne* (2 spp.), *Myersiella* (1 sp.), *Relictivomer* (1 sp.), *Stereocyclops* (2 spp.), *Synapturanus* (3 spp.), *Syncope* (3 spp.), *Otophryne* (2 spp.) and those genera in the subfamily Gastrophryninae are: *Ctenophryne* (2 spp.), *Dasypops* (1 sp.), *Dermatonotus* (1 sp.), *Elachistocleis* (13 spp.), *Gastrophryne* (4 spp.), *Hamptophryne* (1 spp.), *Hypopachus* (4 spp.), *Nelsonophryne* (2 spp.), and *Chiasmocleis* (25 spp.).

While previous phylogenetic analyses [9,11,15,16] have offered much insight regarding microhylid evolution, these studies have included a low number of genera relative to the described levels of diversity (particularly within the NWM). In this paper we present a phylogenetic analysis of microhylid relationships featuring an unprecedented taxonomic sampling with emphasis on NWM diversity and relationships. In addition, we investigated the putative timing of lineage divergence in two ancient microhylid radiations, Gastrophryninae and Otophryninae.

## Methods

### Taxonomic sampling

We used the frequently cited amphibian systematics resource, Amphibian Species of the World [12] as a taxonomic reference for the allocation of genera to subfamilies and to identify those taxa with an *incertae sedis* status within Microhylidae. Focusing on NWM, our sampling within Microhylidae included representatives from 10 of the 11 recognized subfamilies (we did not include the monotypic subfamily Melanobatrachinae; see below for explanation). Microhylid genera included in the analyses were (in parenthesis is the percentage of currently recognized genera that we sampled from each subfamily): *Oreophryne, Austrochaperina, Aphantophryne, Callulops, Choerophryne, Copiula, Cophixalus, Genyophryne, Hylophorbus, Liophryne, Metamagnusia, Sphenophryne, and Xenorhina,* (59 % of Asterophryinae); *Anodontohyla, Platypelis, Plethodontohyla, Rhombophryne,* and *Stumpfia,* (71 % of Cophylinae); *Dyscophus* (100% of Dyscophinae); *Chiasmocleis, Ctenophryne, Dasypops, Dermatonotus, Elachistocleis, Gastrophryne, Hamptophryne, Hypopachus, and Nelsonophryne* (100% of Gastrophryninae); *Hoplophryne* (50% of Hoplophryninae); *Kalophrynus* (100% of Kalophryninae); *Calluella, Chaperina, Glyphoglossus*, *Kaloula, Metaphrynella, Microhyla, Micryletta, Ramanella,* and *Uperodon* (100% of Microhylinae); *Otophryne* (100% of Otophryninae); *Phrynomantis* (100% of Phrynomerinae)*;* and *Scaphiophryne* (50% of Scaphiophryninae). The following genera currently considered *incertae sedis* within Microhylidae [12] were also sampled *Altigius, Arcovomer, Gastrophrynoides, Hyophryne, Melanophryne, Myersiella, Relictivomer, Stereocyclops, Synapturanus,* and *Syncope; Phrynella* sequences from Genbank were included in the analyses*.* In total, our sampling of New World microhylids (i.e., combined Gastrophryninae, Otophryninae, and *incertae sedis* genera), corresponds to 95% of currently recognized genera, missing only *Adelastes*.

We also included 25 other ranoid frogs from families closely related to Microhylidae in our analysis as outgroups. These outgroup taxa were sampled from 8 families and included frogs in the following genera: *Breviceps, Callulina, Probreviceps,* and *Spelaeophryne* (Family Breviciptidae), *Hemisus* (Family Hemisotidae), *Afrixalus*, *Hyperolius*, and *Kassina* (Family Hyperoliidae), *Arthroleptis* and *Leptopelis* (Family Arthroleptidae), *Gephyromantis* (Family Mantellidae), *Ptychadena* (Family Ptychadenidae), *Hylarana and Lithobates* (Family Ranidae), *Polypedates* (Family Rhacophoridae), *Strongylopus* and *Tomopterna* (Family Pyxicephalidae). We used three distantly related outgroups to root our phylogenies: *Xenopus laevis* (Family Pipidae), *Alytes obstetricians* (Family Discoglossidae), and *Scaphiopus holbrooki* (Family Scaphiopodidae). Our global sampling included a combination of our own data (159 taxa; 70%) and DNA sequences downloaded from GenBank (68 taxa; 30%). Genbank accession numbers and voucher information for taxa used in our phylogenetic analyses can be found in Additional file 1; sequences from Genbank are listed in Additional file 2.

### Molecular methodology

Total DNA was isolated from liver or muscle tissue using the Qiagen DNeasy kit (Valencia, California, USA). We used one mitochondrial (16S) and three nuclear (BDNF, tyrosinase, and 28S rRNA) genes. Gene fragments were amplified using previously published primer sets (Table 1). PCRs were conducted using Green or Red Taq polymerase (Promega) and a combination of previously described standard and touchdown thermal cycling profiles that are used to amplify nuclear and mitochondrial DNA from frogs [19,20]. PCR products were cleaned using Ampure magnetic beads (Agencourt® Bioscience, Beverly, Massachusetts, USA) or USB ExoSap-IT (US78201, Ambersham Biosciences, Piscataway, New Jersey, USA) and sequenced (in both primer directions) by SeqWright Corp. (Houston, Texas, USA; http://www.seqwright.com). Resulting chromatograms were visualized and cleaned using the programs Sequencher 5.0 (Gene Codes Corp., Ann Arbor, Michigan, USA). DNA sequences generated for this study were submitted to GenBank; accession numbers are given in Additional file 1.

**Table 1:** Primer sets used for the amplification and sequencing of nuclear (nDNA) and mitochondrial (mtDNA) DNA

**Locus (Primer)**	**Type**	**Direction**	**Sequence (5’ to 3’)**	**Reference**
16S (16SAR)	mtDNA	F	CGCCTGTTTATCAAAAAC AT	[21]
16S (16SBR)	mtDNA	R	CCGGTCTGAACTCAGATCACGT	[21]
28S (28SV)	nDNA	F	AAGGTAGCCAAATGCCTC ATC	[22]
28S (28SJJ)	nDNA	R	AGTAGGGTAAAACTAACC T	[22]
BDNF (BDNF.Amp.F1)	nDNA	F	ACCATCCTTTTCCTTACTATG G	[16]
BDNF (BDNF.Amp.R1)	nDNA	R	CTATCTTCCCCTTTTAATGGTC	[16]
Tyrosinase (TyrC)	nDNA	F	GGCAGAGGAWCRTGCCAAGATGT	[23]
Tyrosinase (TyrG)	nDNA	R	TGCTGGCRTCTCTCCARTCCC A	[23]

### Phylogenetic analyses

Sequence alignments for each locus were initially produced in Sequencher 5.0 or SATé-II [24] using MAFFT aligner and OPAL merger and further modified by eye. For ribosomal subunit genes (28S and 16S) we excluded regions that likely correspond to hyper variable loop regions that were ambiguously aligned (i.e., we removed any regions possessing multiple gapped sites that did not contain readily identifiable sequence motifs). We used the program MacClade 4.08 [25] to infer reading frames for protein coding regions (BDNF, Tyr) and to concatenate the four loci. Our concatenated alignment was deposited in TreeBase (http://www.treebase.org; Study ID: 13478). We only included individuals in our analyses that possessed two or more of the four loci. This criterion excluded the taxon *Melanobatrachus indicus* (Melanobatrachinae) since at present there is only a single locus available that overlaps with our genetic sampling (16S).

Given the size of our dataset, we used the CIPRES gateway server [26] to run parallel versions of several programs including GARLI 1.0[27], PAUPRat [28], BEAST 1.7.2 [29] and MrBayes 3.1.2 [30]. All of these programs were run using machines on the XSEDE server. We also conducted several analyses locally using the program MEGA 5.05 [31]. Collectively our analyses span three widely used phylogenetic criteria for tree searching (Probabilistic: GARLI 1.0, BEAST 1.7.2, and MrBayes 3.1.2; Parsimony: PAUPRat; and Distance: MEGA 3.1.2). For probabilistic analyses, we employed the GTR+I+G model of nucleotide evolution for all genes and partitions since all other substitution models are incorporated within the GTR model [11,32]. Maximum likelihood (ML) analyses were conducted in GARLI 1.0 using default settings and 1000 bootstrap pseudoreplicates (in the form of 20 runs of 50 pseudoreplicates on the XSEDE server). Each GARLI 1.0 analysis invoked a single GTR+I+G model with four gamma categories applied across the entire concatenated dataset. We conducted additional probabilistic analyses by running Bayesian Markov Chain Monte Carlo (BAYES MCMC) simulations in the program MrBayes 3.1.2. These parallel Bayesian analyses were partitioned into eight segments by gene (28S, 16S) and codon position (BDNF and Tyr) using all GTR+I+G models and run for ten million generations with sampling occurring every 1000 generations. We confirmed that each of our MCMC runs had converged by examining the standard deviation of split frequencies and by checking for topological convergence with the online program AWTY [33]. To employ a maximum parsimony (MP) criterion, we conducted 10 searches of 200 iterations each using PAUPRat. Finally, we performed minimum evolution (ME) analyses using 1000 bootstrap pseudorelicates in MEGA 5.05. When necessary, resulting trees from our searches were summarized using TreeAnnotator 1.7.2 and TreeStat 1.7.2 (as implemented in the BEAST software package) and visualized in FigTree 1.3.1 [34].

### Divergence date estimation

To leverage our extensive sampling of NWM (Gastrophryninae + Otophryninae) and to provide a relative temporal framework for patterns recovered during our analyses, we generated a time tree in BEAST 1.7.2. Prior to generating divergence estimates, we pruned the family-scale dataset so that each NWM genus was represented by no more than five nominal member species. Our time tree was calibrated by using three nodal constraints that correspond to: (1—2) the respective origins proposed for Otophryninae and Gastrophryninae [15] and (3) fossil records for *Gastrophryne* from North America [35]. A previous study [15] used two different relaxed clock methods to estimate dates [36,37]; in their study their estimates (across both methodologies) ranged from 51.7 to 69.1 mya for the origin of Otophryninae and 66.8 to 91.4 mya for the origin of Gastrophryninae. To use these hypotheses of divergence, we took the mean of each estimate (60.4 mya, Otophryninae; 79.1 mya, Gastrophryninae) and by using a normal distribution with 5 standard deviations constrained these nodes to the approximate ranges reported before [15]. A similar strategy was employed to incorporate the ca. 1.7 my old series of *Gastrophryne* fossils [35,38] by using a normal distribution with 0.5 standard deviations to constrain the node leading to *G. carolinensis*, *G. olivacea*, and *G. mazatlanensis* as having occurred between 0.72 and 2.68 mya. This calibration point was used because several *G. carolinensis*, *G. olivacea*, and *G. mazatlanensis* fossils have been reported from Pleistocene deposits ranging in age from 0.24 to 1.8 mya [38]. We employed a lognormal relaxed clock and a Yule speciation prior [39] to estimate trees and divergence dates in a Bayesian MCMC run featuring a chain length of ten millions with sampling occurring every 1000 generations. We partitioned our dataset by gene and applied unlinked GTR+I+G models with 4 gamma rate categories to each of the 4 partitions. We used Tracer 1.5 [40] to view the BEAST 1.7.2 output and identify that all parameters were adequately sampled (i.e., ESS > 200). A burn-in of 1000 was used prior to summarizing time trees.

## Results and discussion

### Molecular analysis

Our family level data matrix consisted of 225 taxa, and 2673 base pairs (BDNF [711 bp], Tyr [551 bp], 28S [738 bp], and 16S [673 bp]). This concatenated dataset contained 938 parsimony informative characters, 239 uninformative variable characters, and 1496 constant (invariant) characters. The amount of phylogenetic information was variable across loci (number of parsimony informative sites/total sites): Tyr (313/551, 60%), BDNF (221/711, 31%), 28S (49/738, 6.6%), and 16S (355/673, 52%). The results of our phylogenetic analyses were largely consistent with previous studies. This is particularly encouraging given that our study included fewer nucleotide characters than either of those studies [15,40]. Our ML data matrix consisted of 361,442 unique patterns and resulted in a topology with a log likelihood score of −46681.5016. We recovered almost identical topologies from the ML and Bayesian MCMC searches with most variation confined to the internal composition of tip groups. The examination of topological convergence (AWTY analysis) between our parallel Bayesian searches revealed that while the analyses did not converge, at around five million generations they stabilized at approximately 2 symmetric differences from one another. Subsequent examination of consensus trees from each run revealed few differences, so we derived posterior probabilities from these 10,000 tree sets using a burn-in of 5000 samples. Our tree searches that employed a parsimony ratcheting approach recovered largely concordant patterns with Bayesian and likelihood analyses. We were required to remove 15 taxa from our alignment in order to conduct the distance-based (ME) analyses because pair-wise estimates could not be generated due to missing data. While the resulting ME searches featured topologies with broadly consistent patterns relative to the parsimony and probabilistic analyses, we recovered weak nodal support for most groupings beyond shallow phylogenetic depths and several alternative arrangements of taxa relative to the MP, ML, and BAYES MCMC analyses. We do not, however, interpret these inconsistencies as meaningful given the known effects of missing character information on distance-based criteria and the variable genetic sampling strategy we employed [41,42]. The placement of samples obtained from GenBank, e.g., *Copiula* sp. [GB] and *Cophixalus* sp. [GB] suggests that these taxa may have been misidentified in previous studies.

**Figure 2:**
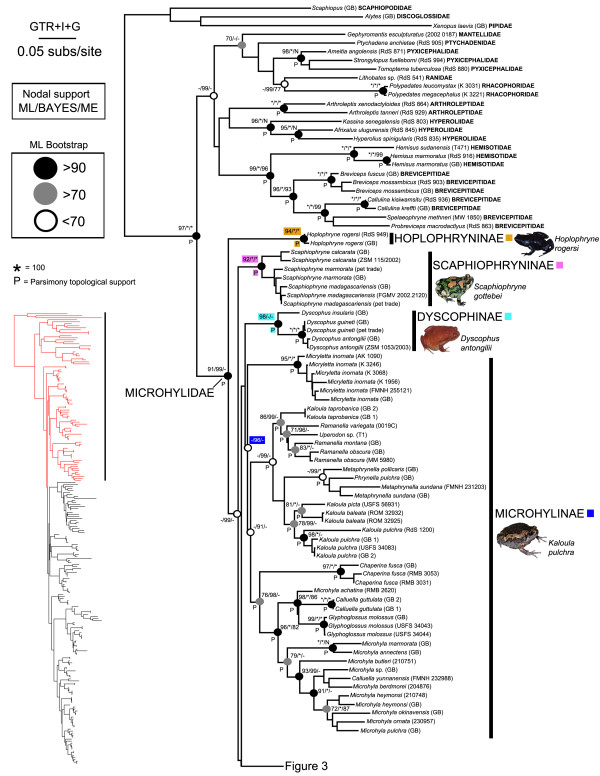
**Maximum likelihood phylogram depicting relationships between microhylid taxa sampled for this study.** Nodal support values above nodes correspond to ML bootstrapping, BAYES MCMC posterior probabilities, and ME bootstrapping respectively. * = value of 100, P = clade also recovered by MP PAUPRat analysis, GB = DNA sequences from GenBank (Additional file 2); see also Figures 3 and 4 .

**Figure 3:**
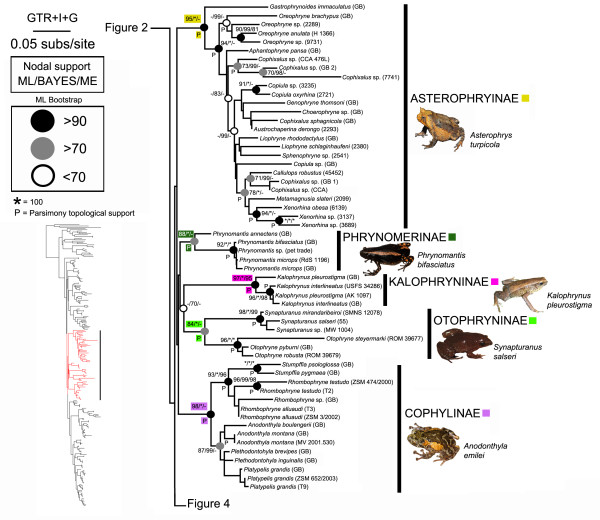
**Maximum likelihood phylogram depicting relationships between microhylid taxa sampled for this study.** Nodal support values above nodes correspond to ML bootstrapping, BAYES MCMC posterior probabilities, and ME bootstrapping respectively. * = value of 100, P = clade also recovered by MP PAUPRat analysis, GB = DNA sequences from GenBank (Additional file 2); see also Figures 2 and 4 .

**Figure 4:**
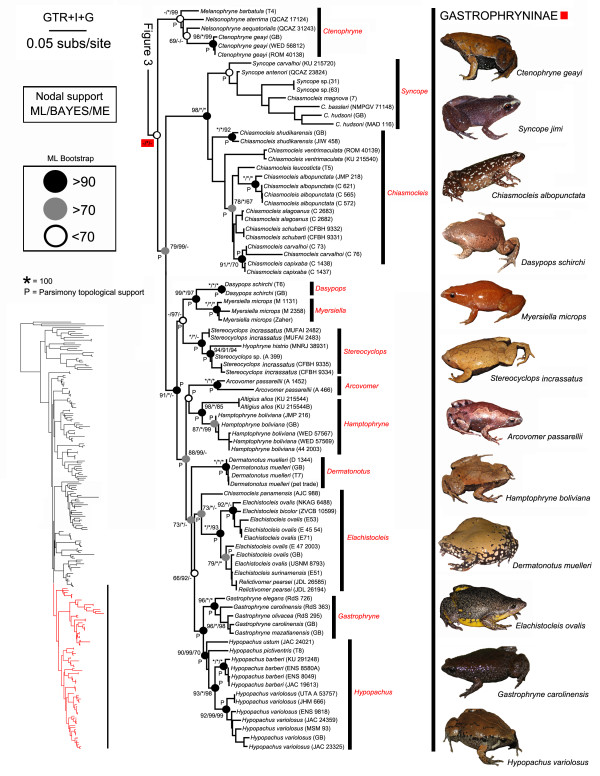
**Maximum likelihood phylogram depicting relationships within the subfamily Gastrophryninae.** Nodal support values above nodes correspond to ML bootstrapping, BAYES MCMC posterior probabilities, and ME bootstrapping respectively. * = value of 100, P = clade also recovered by MP PAUPRat analysis, GB = DNA sequences from GenBank (Additional file 2); see also Figures 2 and 3 .

Below, we summarize our phylogenetic results based on the ML tree (Figures 2, 3, 4) in relation to (1) *incertae sedis* genera and (2) microhylid subfamilies. Bootstrap support values of 70% or higher were considered to be relatively strong nodal support [43]; clades that were topologically supported in the parsimony analysis are indicated in Figures 2, 3, 4 with a “P”.

### *Genera currently placed as* incertae sedis

With the single exception of *Adelastes hylonomos* our analyses included all *incertae sedis* genera currently placed in Microhylidae [12]. Regarding *incertae sedis* taxa originating from the Old World, we recovered *Gastrophrynoides* as a basal member of the Asterophryinae and *Phrynella* nested within *Metaphrynella* in the subfamily Microhylinae (Figures 2, 3). These findings are in overall agreement with a recent study, except that *Phrynella* was previously found as the sister taxon to *Metaphrynella*[44]. Consequently, herein we place *Gastrophrynoides* in the Asterophryinae and *Phrynella* in the Microhylinae.

All of the *incertae sedis* genera we sampled from the New World were placed within the Gastrophryninae by our analyses, except *Synapturanus* that was recovered as the sister taxon to *Otophryne* (Figure 4). The high-altitude *Melanophryne* was consistently placed in a clade with *Nelsonophryne* and *Ctenophryne*, although the relationships among these genera varied. The clade containing these three genera is the sister group to all other gastrophrynines. The genus *Chiasmocleis* as currently recognized is polyphyletic consisting of three distinct groups 1) *Chiasmocleis panamensis* (which is more closely related to *Elachistocleis* than other *Chiasmocleis* species), 2) a clade consisting of three species of *Chiasmocleis* nested in *Syncope*, and 3) all other species of *Chiasmocleis*. *Relictivomer* is nested within *Elachistocleis*; *Dasypops* is the sister taxon to *Myersiella* and these two genera share a sister relationship with *Stereocyclops*; *Hyophryne* is nested within *Stereocyclops*; and *Arcovomer* is sister to a clade containing *Altigius* and *Hamptophryne*.

### Relationships among Microhylid subfamilies

The monophyly of Microhylidae is strongly supported on the basis of morphology [8,45] and molecules [9,11], this study). Additionally, the existence and content of 11 major microhylid evolutionary lineages (i.e., subfamilies) is nearing a consensus [9,11,15,16], this study]. The relationship of these subfamilies to one another, however, remains enigmatic with each available dataset recovering a slightly different phylogenetic arrangement at this deep evolutionary tier. The poor resolution of inter familial-relationships is likely to be related to the short amount of evolutionary time that separated the origin of each major group during the late Cretaceous [15]. While our analysis did not recover branch support for inter-familial relationships, below we discuss the similarities and differences between our results and those of the four previous studies that sampled microhylids at this phylogenetic depth [9,11,15,16].

Our analyses produced strong support for the reciprocal monophyly of eight of the ten subfamilies we sampled. Although Microhylinae and Gastrophryninae did not receive nodal support in our bootstrapping analyses, these two subfamilies were monophyletic in the ML tree and received strong support from the Bayesian analyses (see Figures 2, 3, 4). Hoplophryninae and Scaphiophryninae were recovered as the earliest branches on the microhylid tree, followed by two major clades consisting of the remaining subfamilies [branching order in brackets]: (1) Gastrophryninae, Asterophryinae, Cophylinae, Phrynomerinae, Otophryninae, and Kalophryninae {Gastrophryninae [Asterophryinae (Cophylinae (Phrynomerinae (Otophryninae-Kalophryninae)))]} and (2) Microhylinae and Dyscophinae (Figures 1, 2, 3, 4).

In contrast to our analyses, other studies recovered the most basal lineages as: Phrynomerinae [11,15,44], Phrynomerinae-Gastrophryninae [16], or *Kalophrynus* (*Synapturanus* (*PhrynomantisMicryletta*))] [9]. The somewhat basal position of Scaphiophryninae in our analyses has not been suggested previously; this taxon was found either closely related to Microhylinae [9] or Cophylinae [16]. The close relationship between Dyscophinae and Microhylinae has been suggested before, but with alternative sister relationships to either Asterophryinae [15,16,44] or to (Kalophryninae-(Melanobatrachinae-Asterophryinae)) [11]. An arrangement in which Microhylinae is closely related to Scaphiophryninae and Dyscophinae to Asterophryinae has also been suggested [9].

The second, and largest, clade recovered consists of the remaining subfamilies: 1) a basal Gastrophryninae, 2) Asterophryinae basal to the remaining subfamilies, and 3) Cophylinae basal to a clade consisting of [Phrynomerinae (Otophryninae-Kalophryninae)]. Previous analyses recovered a Phrynomerinae basal to all microhylids [11,15,16]; *Phrynomantis* was considered *incertae sedis*[9]. Kalophryninae or Otophryninae were not sampled [16,44] and Kalophryninae was recovered in a clade with Cophylinae and Melanobatrachinae [15] or in a clade with Melanobatrachinae and Asterophryinae [11].

Gastrophryninae has been reported to have a variety of phylogenetic affinities including: 1) a sister relationship with Cophylinae [9,44], 2) basal to all microhylids excluding *Synapturanus*, *Scaphiophryne*, *Hoplophryne*, and *Phrynomantis*[15], 3) a sister relationships with Phrynomerinae [16], and 4) within a monophyletic clade containing Hoplophryninae and Cophylinae that is basal to all other subfamilies excluding Phrynomerinae and Otophryninae [11].

Given the amount of instability regarding these sub-familial relationships across different studies, we feel that any tenable phylogenetic hypothesis of their relatedness will await additional genetic sampling. However, it is interesting to note that using an almost independent data set we recovered patterns indicative of rapid and early diversification in microhylids that are consistent with previous studies [15].

### Relationships within Old world subfamilies

The content and phylogenetic arrangement of taxa within Hoplophryninae, Scaphiophryninae, Dyscophinae, Phrynomerinae, and Kalophryninae was consistent with previous analyses. Within Microhylinae we recovered three major clades consisting of: 1) the widespread *Micryletta inornata* complex, 2) *Ramanella*, *Uperodon*, *Kaloula*, *Phrynella*, and *Metaphrynella*, and 3) *Chaperina*, *Microhyla*, *Calluella*, and *Glyphoglossus*. The content of these clades is broadly consistent with previous molecular studies [46]. Within our sampling of this subfamily four genera appear to be paraphyletic: *Kaloula*, *Microhyla*, *Calluella*, and *Ramanella*. Previous research suggests that levels of diversity within the subfamily Asterophryninae are staggering [47]. We employed a sampling strategy to maximize our taxonomic coverage (i.e., we selected evolutionarily distinct lineages based on previous mtDNA studies). Given the phylogenetic depth and diversity within this group, our strategy resulted in many long branches and weakly supported nodes. While our commentary on relationships within this subfamily is limited, as previously reported [47] it seems likely that the genera *Copiula*, *Callulops*, *Cophixalus*, and *Liophryne* are paraphyletic taxa. Our analyses were consistent with previous studies in the clustering of some *Liophryne* species and *Sphenophryne*, a monophyletic *Oreophryne*, and a monophyletic *Xenorhina*. As was observed in the original description [48], our trees placed *Metamagnusia* as a close relative of *Xenorhina*. Within the Cophylinae the relationships that we recovered are very similar to those reported in a previous molecular study [49].

### Relationships among New world Genera and taxonomic implications

We recovered a close relationship between *Otophryne* and *Synapturanus* and therefore we agree with the recent placement of *Synapturanus* in this subfamily [11]. In light of all available studies that included *Otophryne* and/or *Synapturanus* and our analyses that included all other New World genera (except *Adelastes*), it is likely that Otophryninae is more closely related to other microhylid subfamilies than it is to the sympatric subfamily Gastrophryninae. The distinctiveness of *Otophryne* from all other NWM was noted earlier based on the following unique combination of morphological characters: omosternum present, clavicles straight, and a well-developed tympanum [50]. Furthermore, this author indicated that elsewhere in the Microhylidae this combination is only found in the genus *Kalophrynus* (member of the Asian subfamily Kalophryninae)*.* A similar relationship between *Kalophrynus* and *Otophryninae* was observed in a family-level morphological analysis [5]. Interestingly, our family-level analysis also recovered Kalophryninae and Otophryninae as sister taxa, although with weak nodal support (Figure 2).

Because NWM are not a monophyletic assemblage, previous morphological studies that assessed relationships among NWM and included *Otophryne* and/or *Synapturanus* along with Gastrophryninae genera need to be reassessed since morphological and karyological similarities between Otophryninae and Gastrophryninae are either primitive characters present in both lineages or homoplasies resulting from parallel or convergent evolution. Below, we suggest several taxonomic changes within the Gastrophryninae to better reflect the evolutionary history of this subfamily (Figure 4).

### *Ctenophryne*, *Melanophryne*, and *Nelsonophryne*

The most recently described genus of NWM, *Melanophryne* Lehr and Trueb, 2007, forms a monophyletic group with *Nelsonophryne* Frost, 1987 and *Ctenophryne* Mocquard, 1904. Our phylogenetic analyses recovered variable patterns of relatedness among these genera (see support values in Figure 4). Our ME analysis recovered a monophyletic *Ctenophryne* and *Melanophryne* nested within *Nelsonophryne*. In the ML analysis, *Melanophryne* is basal to the entire *Ctenophryne-Nelsonophryne* clade, whereas in the Parsimony and Bayesian MCMC topologies *N. aterrima* has a basal position and *Melanophryne* is closer to a clade consisting of *CtenophryneN. aequatorialis*. The presence of a maxilla-quadratojugal articulation in *Ctenophryne* and *N. aequatorialis* and its absence in *N. aterrima* was recently reported [6]. Potential morphological differences between *Nelsonophryne* and *Ctenophryne* are: *Nelsonophryne* has neopalatines whereas *Ctenophryne* lacks them [51,52] and distal carpals 3—5 fuse in *Ctenophryne* whereas only 4—5 fused in *Nelsonophryne*[6]. Undoubtedly, this clade needs further study and we suspect that additional species will be discovered and relationships will need further assessment. However, given the shallow phylogenetic depth of the *Ctenophryne*+*Melanophryne*+*Nelsonophryne* clade and to tentatively resolve the paraphyly of *Nelsonophryne*, we place *Nelsonophryne* Frost, 1987 and *Melanophryne* Lehr and Trueb, 2007 in the synonymy of *Ctenophryne* Mocquard, 1904, which produces the new taxonomic combinations *Ctenophryne aequatorialis* (Peracca, 1904), *Ctenophryne aterrima* (Günther, 1901), *Ctenophryne barbatula* (Lehr and Trueb, 2002), and *Ctenophryne carpish* (Lehr, Rodríguez, and Córdova, 2007).

Described larvae for this clade are: *Ctenophryne aterrima*[53], *C. aequatorialis*, *C. carpish*[54], *and C. gaeyi*[55].

### *Chiasmocleis* and *Syncope*

The genus *Syncope* Walker, 1973 was recovered in a clade with *Chiasmocleis bassleri*, *C. hudsoni*, and *C. magnova,* rendering *Chiasmocleis* Mehely, 1904 paraphyletic. There are two alternative solutions to resolve this paraphyly: 1) synonymize *Syncope* with *Chiasmocleis* or 2) recognize *Syncope* as a separate evolutionary lineage and transfer some currently recognized species of *Chiasmocleis* to *Syncope*. We opted for the second alternative to recognize the separate evolutionary trajectory of this lineage based on shared morphological and life history traits. Zweifel [56]:21] suggested the possibility of a close relationship between *Syncope* and some *Chiasmocleis* species based on digital reduction. *Syncope* currently consists of three species and, in terms of overall body size, it contains the smallest species of gastrophrynine microhylids. Furthermore, *Syncope* species have lost two vertebrae and have reduced and/or lost fingers I and IV. A similar pattern of small adult body size and digit reduction is present in the species of *Chiasmocleis* that we found to share phylogenetic affinities with *Syncope*: *Chiasmocleis bassleri, C. hudsoni,* and *C. magnova*. Other *Chiasmocleis* (apart from *C*. *jimi* and *C*. *supercilialbus*[57,58]) do not show reduction in adult body size and/or the number of digits. A life history trait that may further unite *Syncope* with the small *Chiasmocleis* species is their reproductive mode. *Syncope antenori* was thought to have direct-development based on large eggs and small clutch sizes [59,60]. However, this taxon was later shown to have free-swimming, endotrophic larvae that develop in water-filled bromeliads [60]. The original description of *C. magnova* also suggested that the species might be a direct developer [58], based mainly on the presence of large eggs in the oviducts of the holotype. Thus, based on egg size, *S. antenori* and *C. magnova* may have similar reproductive modes. Herein, we place the following species of *Chiasmocleis* in the genus *Syncope* which produces the new taxonomic combinations *S. bassleri* (Dunn 1949), *S. hudsoni* (Parker, 1940) and *S. magnova* (Moravec and Köhler, 2007) (based on our phylogeny) and *S. jimi* and *S. supercilialbus* based on the morphological description of the species (‘…first toe reduced,’ [57]:2]) and (‘…fingers I and IV reduced…’ [58]:60]). This new taxonomic re-arrangement renders *Chiasmocleis* Mehely, 1904 monophyletic (with exception to *C. panamensis*; see below) and expands the content of *Syncope* Walker, 1973. Furthermore, this taxonomic arrangement recognizes the unique morphological patterns (i.e., a trend toward smaller adult body size and reduction and loss of vertebrae and/or digits in the forelimbs)and specialized life history traits in *Syncope*. It is also consistent with morphological variation in the pectoral girdle where there has been a complete loss of the connection between coracoids and epicoracoid in *S. antenori* and *S. magnova* (and a reduced connection in *S. jimi* and *S. hudsoni*) whereas the connection is present in *Chiasmocleis*[61].

Free-swimming larvae have been reported for *Chiasmocleis alagoanus*[62], *C. albopunctata*[63], *C. anatipes*[64], *C. carvalhoi*[65], *C. leucosticta*[66], *C. mantiqueira*[67], *C. shudikarensis*[68], and *C. ventrimaculata*[55,69]. Description of *Syncope* larvae is limited to *S. antenori*[60] and *S. hudsoni*[69].

Another problematic species is *Chiasmocleis panamensis* that was not recovered within *Chiasmocleis* or *Syncope*, but rather as the sister taxon of the genus *Elachistocleis; a* relationship recovered with robust support in all our analyses*.* Therefore, we place *C. panamensis* in the genus *Elachistocleis* that produces the new taxonomic combination *Elachistocleis panamensis* (Dunn et al., 1948). The phylogenetic placement *E. panamensis* is not surprising given that 1) the original description of species includes the following statement: “… Dunn was quite dubious as to their identity but thought they might be *Elachistocleis*, at that time the only microhylid recorded from Panama” [70]:1] and 2) a previous morphological analysis placed this taxon outside of *Chiasmocleis,* although not closely related to *Elachistocleis*[61].

### *Stereocyclops* and *Hyophryne*

The Bahia yellow frog, *Hyophryne histrio* Carvalho, 1954, was consistently recovered as nested within *Stereocyclops* (Figure 4). Consequently, we place the monotypic *Hyophryne* in the synonymy of *Stereocyclops* Cope 1870. This arrangement produces the new taxonomic combination: *Stereocyclops histrio* (Carvalho, 1954).

*Hyophryne* was considered morphologically related to *Stereocyclops* and the two genera were separated based on characteristics of the pectoral girdle, particularly a long clavicle and a reduced procoracoid in *Stereocyclops* and short clavicle and long procoracoid in *Hyophryne*[51]. However, a recent study showed the procoracoid to be highly variable in *Hyoprhyne*[71]. *Hyophryne* has been included only in two other studies [53,56]. One study [56] found no diagnostic characters to separate *Hyophryne* from *Stereocyclops* and the author indicated that “…nonmorphological data on *Hyophryne* (it is known only from the holotype) should help define its position.” A study that assessed the relationships of *Altigius* to putative relatives recovered *Hyophryne* closely related to *Hamptophryne*[72]. Most recently, a study provided a detailed analysis of *Hyophryne* that significantly increased our understanding of the morphology and biology of this poorly known genus [71]; the author concluded that *Hyophryne* was the sister taxon of *Stereocyclops*.

The larva of *S. histrio* is unknown whereas descriptions are available for *S. incrassatus*[73,74] and *S. parkeri*[74].

### *Arcovomer*, *Altigius*, and *Hamptophryne*

In our consensus topology, *Arcovomer passarellii* Carvalho, 1954 is most closely related to *Altigius alios* Wild, 1995 and *Hamptophryne boliviana* (Parker, 1927). Currently, all three of these genera are monotypic. However, two new species of *Arcovomer* from Brazil, one from central-north São Paulo and the other one from Espírito Santo, are being described by one of us (CFBH). Given the close phylogenetic relationship between *Altigius* and *Hamptophryne*, we place the genus *Altigius* in the synonymy of *Hamptophryne* Carvalho, 1954 which produces the new taxonomic combination *Hamptophryne alios* (Wild, 1995). A close affinity between *Arcovomer* and *Hamptophryne* was previously suggested [51] based on both genera lacking neopalatines and having divided prevomers. The condition of the posterior vomers has been reported to vary in this clade with *H. boliviana* possessing posterior vomers reduced to small plates and *Arcovomer* possessing these elements as a fused single element found anterior to the parasphenoid. Osteological information for *H. alios* is very limited and incomplete but the original description indicates “…posterior vomer and neopalatines not distinguishable” [72].

Descriptions of larvae within this clade are available for *H. alios*[72] and *H. boliviana*[64]. The larva of *Arcovomer* has not been described.

### *Dasypops* and *Myersiella*

We recovered strong support for a sister relationship between the genera *Myersiella* Carvalho, 1954, and *Dasypops* Miranda-Ribeiro, 1924 (Figure 4). While both of these genera are currently monophyletic, at least one new species of *Myersiella* from Minas Gerais, Brazil, is being described by one of us (CFBH). These genera are similar in having small heads relative to total body size; in *Dasypops* the snout is broad and truncated whereas it is narrow and pointed in *Myersiella*[56]. These genera can be differentiated by 1) the fingers and toes which are swollen in *Dasypops* and slender in *Myersiella*[75] and 2) presence of clavicle and procoracoid in *Dasypops* but absent in *Myersiella*[51]. Herein, we note some additional differences between those two genera: 1) the condition of finger IV which is comprised of two phalanges in *Dasypops* and three in *Myersiella*, 2) a broad parasphenoid that extends beyond the choanae in *Dasypops* and a slender and not reaching the choanae in *Myersiella*, 3) the advertisement call which is trilled in *Dasypops* and consists of simple whistles [76] in *Myersiella*[77], and 4) aquatic and free-swimming larvae in *Dasypops*[77] and direct-development in *Myersiella*[78]. The phylogenetic placement of *Myersiella* deep within the Gastrophryninae may represent a notable instance of convergence given the morphological [56], behavioral, and reproductive [79] characteristics it shares with the otophrynine *Synapturanus*.

### *Dermatonotus, Elachistocleis*, *Relictivomer, Gastrophryne,* and *Hypopachus*

The monotypic *Dermatonotus* Mehely, 1904 is sister to a clade that includes *Elachistocleis*, *Gastrophryne* Fitzinger, 1843 and *Hypopachus* Keferstein, 1867. *Dermatonotus* was proposed to be ‘allied’ with *Hypopachus* and *Gastrophryne*[51]; furthermore Carvalho suggested that the genus might be “….close to the ancestral stock that gave rise to *Nelsonophryne* (= Glossostoma; *sensu* Günter, 1901), *Hypopachus*, *Gastrophryne*, *Relictivomer*, *Elachistocleis*, *Dasypops*, *Myersiella*, and *Synapturanus*.” Also, a close association among *NelsonophryneGlossostoma]*, *Hypopachus*, *Gastrophryne*, and *Elachistocleis* was suggested previously [76,80]. Our results agree with the previous suggestion that *Dermatonotus* is basal to several genera: *Hypopachus*, *Gastrophryne*, *Elachistocleis*, and *Relictivomer*. However, *Dasypops*, *Myersiella*, and *Ctenophryne* (including ‘*Glossostoma’*) appear to have resulted from earlier branching events in the Gastrophryninae tree than *Dermatonotus*. The phylogenetic patterns that we recovered for *Dermatonotus*, *Elachistocleis*, *Gastrophryne*, and *Hypopachus* are generally congruent with previous molecular studies [11,16,17], although *Dermatonotus* was not included in the latter study. While *Dermatonotus* is presently considered to be monotypic it is likely to represent a complex of species distributed from the Chaco of Argentina to Bolivia, Paraguay and reaching northeastern Brazil (Maranhão State). Furthermore, a second species is being described from Northeastern Brazil by one of us (CFBH).

Our analyses recovered the monotypic genus *Relictivomer* nested within a well-supported clade of *Elachistocleis* samples (Figure 4). A close relationship between these genera was previously suspected on the basis of morphology [61]. *Relictivomer* was differentiated from *Elachistocleis*[51] based on the presence of reduced posterior vomers in the former and their absence in the latter genus. Based on our phylogeny, we return *R. pearsei* (Ruthven, 1914) to the genus *Elachistocleis* Parker, 1927 resurrecting the taxonomic combination *Elachistocleis pearsei*. Adult *Elachistocleis*, including *E. pearsei*, have the following combination of characters: clavicle short and curved, distal end of the clavicle curved not touching the coracoid, procoracoid divided, and the last three vertebrae longer than wide. The condition of the last three vertebrae is a putative synapomorphy for *Elachistocleis*. While this state in adult *E. panamensis* awaits confirmation, a juvenile specimen exhibited wider than long vertebrae; the juvenile condition could imply the retention of the ancestral state in this early branching lineage of *Elachistocleis* or that the last three vertebrae grow postmetamorphically becoming longer than wider in adults.

Our analyses also support the recent placement of *Gastrophryne usta* and *G. pictiventris* in *Hypopachus*[18]. However, we recovered a different phylogenetic arrangement among members of the genus *Gastrophryne*. Previous authors hypothesized that *G. elegans* and *G. olivacea* are sister taxa, our analyses exclusively grouped *G. carolinensis* and *G. olivacea* (as previously suggested [80]). However, our analyses recovered a paraphyletic *G. carolinensis* with respect to *G. olivacea* and *G. mazatlanensis*. Though it warrants further exploration, this enigmatic result may be related to the regular hybridization that occurs between *G. carolinensis* and *G. olivacea*[18] and our molecular sampling strategy that was biased towards nuclear DNA.

Free swimming larvae for this clade have been described for: *Dermatonotus*[81-83], *Elachistocleis bicolor*[73], *E. ovalis*[84], *E. panamensis*[85], *E. pearsi*[86], *E*. *surinamensis*[87], *Gastrophryne carolinensis*[88,89], *G. elegans*[90], *G. olivacea*[89], *Hypopachus barberi*[91,92], *H. pictiventris*[53], *H. ustum*[90], and *H. variolosus*[89,93].

A summary of proposed taxonomic changes is provided in Table 2.

**Table 2:** Redefined content of the subfamily Gastrophryninae with proposed taxonomic modifications (bold text), original subfamily designations, and larval description citations by taxon

**Old taxonomy**	**New taxonomy**	**Original placement**	**Larval description**
*Ctenophryne geayi*	*Ctenophryne geayi*	Gastrophryninae	[56]
*Ctenophryne minor*	*Ctenophryne minor*	Gastrophryninae	None
*Nelsonophryne aequatorialis*	***Ctenophryne aequatorialis***	Gastrophryninae	[54]
*Nelsonophryne aterrima*	***Ctenophryne aterrima***	Gastrophryninae	[53]
*Melanophryne barbatula*	***Ctenoprhyne barbatula***	*incertae sedis*	None
*Melanophryne carpish*	***Ctenophryne carpish***	*incertae sedis*	[54]
*Syncope antenori*	*Syncope antenori*	*incertae sedis*	[68]
*Syncope carvalhoi*	*Syncope carvalhoi*	*incertae sedis*	None
*Syncope tridactyla*	*Syncope tridactyla*	*incertae sedis*	None
*Chiasmocleis bassleri*	***Syncope bassleri***	Gastrophryninae	None
*Chiasmocleis hudsoni*	***Syncope hudsoni***	Gastrophryninae	None
*Chiasmocleis jimi*	***Syncope jimi***	Gastrophryninae	None
*Chiasmocleis magnova*	***Syncope magnova***	Gastrophryninae	None
*Chiasmocleis supercilialbus*	***Syncope supercilialbus***	Gastrophryninae	None
*Chiasmocleis alagoanus*	*Chiasmocleis alagoanus*	Gastrophryninae	[62]
*Chaismocleis albopunctata*	*Chaismocleis albopunctata*	Gastrophryninae	[71]
*Chasimocleis anatipes*	*Chasimocleis anatipes*	Gastrophryninae	[47]
*Chiasmocleis atlantica*	*Chiasmocleis atlantica*	Gastrophryninae	None
*Chiasmocleis avilapiresae*	*Chiasmocleis avilapiresae*	Gastrophryninae	None
*Chiasmocleis capixaba*	*Chiasmocleis capixaba*	Gastrophryninae	None
*Chiasmocleis carvalhoi*	*Chiasmocleis carvalhoi*	Gastrophryninae	[72]
*Chiasmocleis centralis*	*Chiasmocleis centralis*	Gastrophryninae	None
*Chiasmocleis cordeiroi*	*Chiasmocleis cordeiroi*	Gastrophryninae	None
*Chiasmocleis crucis*	*Chiasmocleis crucis*	Gastrophryninae	None
*Chiasmocleis devriesi*	*Chiasmocleis devriesi*	Gastrophryninae	None
*Chiasmocleis gnoma*	*Chiasmocleis gnoma*	Gastrophryninae	None
*Chiasmocleis hudsoni*	*Chiasmocleis hudsoni*	Gastrophryninae	[73]
*Chiasmocleis leucosticta*	*Chiasmocleis leucosticta*	Gastrophryninae	[74]
*Chiasmocleis mantiqueira*	*Chiasmocleis mantiqueira*	Gastrophryninae	[75]
*Chiasmocleis mehelyi*	*Chiasmocleis mehelyi*	Gastrophryninae	None
*Chiasmocleis sapiranga*	*Chiasmocleis sapiranga*	Gastrophryninae	None
*Chiasmocleis schubarti*	*Chiasmocleis schubarti*	Gastrophryninae	None
*Chiasmocleis shudikarensis*	*Chiasmocleis shudikarensis*	Gastrophryninae	[76]
*Chiasmocleis ventrimaculata*	*Chiasmocleis ventrimaculata*	Gastrophryninae	[56,76]
*Hyophryne histrio*	***Stereocyclops histrio***	*incertae sedis*	None
*Stereocyclops incrassatus*	*Stereocyclops incrassatus*	*incertae sedis*	[49,50]
*Stereocyclops parkeri*	*Stereocyclops parkeri*	*incertae sedis*	[50]
*Arcovomer passarellii*	*Arcovomer passarellii*	*incertae sedis*	None
*Altigius alios*	***Hamptophryne alios***	*incertae sedis*	[46]
*Hamptophryne boliviana*	*Hamptophryne boliviana*	Gastrophryninae	[47]
*Dasypops schirchi*	*Dasypops schirchi*	Gastrophryninae	[59]
*Myersiella microps*	*Myersiella microps*	*incertae sedis*	[60]
*Dermatonotus muelleri*	*Dermatonotus muelleri*	Gastrophryninae	[76,80,81]
*Chiasmocleis panamensis*	***Elachistocleis panamensis***	Gastrophryninae	None
*Relictivomer pearsei*	***Elachistocleis pearsei***	Gastrophryninae	[64]
*Elachistocleis bicolor*	*Elachistocleis bicolor*	Gastrophryninae	[49]
*Elachistocleis bumbameuboi*	*Elachistocleis bumbameuboi*	Gastrophryninae	None
*Elachistocleis carvalhoi*	*Elachistocleis carvalhoi*	Gastrophryninae	None
*Elachistocleis cesarii*	*Elachistocleis cesarii*	Gastrophryninae	None
*Elachistocleis erythrogaster*	*Elachistocleis erythrogaster*	Gastrophryninae	None
*Elachistocleis helianneae*	*Elachistocleis helianneae*	Gastrophryninae	None
*Elachistocleis magnus*	*Elachistocleis magnus*	Gastrophryninae	None
*Elachistocleis matogrosso*	*Elachistocleis matogrosso*	Gastrophryninae	None
*Elachistocleis ovalis*	*Elachistocleis ovalis*	Gastrophryninae	[62]
*Elachistocleis skotogastor*	*Elachistocleis skotogastor*	Gastrophryninae	None
*Elachistocleis surinamensis*	*Elachistocleis surinamensis*	Gastrophryninae	[65]
*Elachistocleis surumu*	*Elachistocleis surumu*	Gastrophryninae	None
*Gastrophryne carolinensis*	*Gastrophryne carolinensis*	Gastrophryninae	[82,83]
*Gastrophryne elegans*	*Gastrophryne elegans*	Gastrophryninae	[84]
*Gastrophryne olivacea*	*Gastrophryne olivacea*	Gastrophryninae	[83]
*Gastorphryne mazatlanensis*	*Gastorphryne mazatlanensis*	Gastrophryninae	None
*Hypopachus barberi*	*Hypopachus barberi*	Gastrophryninae	[85,86]
*Hypopachus pictiventris*	*Hypopachus pictiventris*	Gastrophryninae	[53]
*Hypopachus ustum*	*Hypopachus ustum*	Gastrophryninae	[84]
*Hypopachus variolosus*	*Hypopachus variolosus*	Gastrophryninae	[83,87]

Proposed taxonomic modifications are indicated by bold text.

### Divergence dating implications

**Figure 5:**
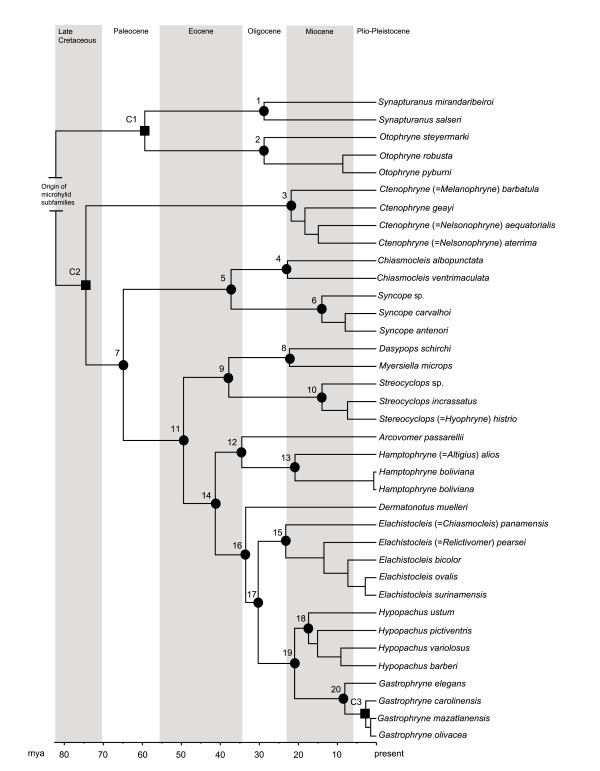
**Bayesian time tree generated from partitioned mitochondrial and nuclear dataset.** Nodes indicated by solid circles correspond to dates listed in Table 3. Calibration points (C1—C3; see text for more details) are indicated as solid squares.

After reducing our taxonomic sampling for the divergence analysis, the multilocus alignment contained 37 taxa and 2683 bp. This dataset produced the time tree depicted in Figure 5. For descriptive purposes we defined several Gastrophryninae subclades (see Table 3): (1) *Chiasmocleis* + *Syncope* + *Dasypops* + *Myersiella* + *Stereocylops* + *Arcovomer* + *Hamptophryne* + *Dermatonotus* + *Elachistocleis* + *Gastrophryne* + *Hypopachus*, (2) *Dasypops* + *Myersiella* + *Stereocylops* + *Arcovomer* + *Hamptophryne* + *Dermatonotus* + *Elachistocleis* + *Gastrophryne* + *Hypopachus*, and (3) *Dermatonotus* + *Elachistocleis* + *Gastrophryne* + *Hypopachus*. The distribution and diversification of the Microhylidae has been associated to the breakup and subsequent drifting of Gondwanaland continents [94]. However, a recent work suggested that the diversification of the microhylid clade occurred during the late Cretaceous [15], after the breakup of Gondwanaland. Consequently, these authors suggest the possibility of land bridge connections among the drifting continents that would have allowed for the dispersal of early microhylid lineages. This biogeographic scenario is supported by: 1) an Otophryninae clade that is more closely related to geographically distant microhylids lineages (e.g., Kalophryninae) than to other NWM [this study] and 2) the correlation between patterns of diversification in Late Cretaceous microhylid lineages and other co-distributed anuran lineages [15].

**Table 3:** **Divergence time estimates in millions of years ago (with 95% highest posterior density [HPD] range) for major nodes (Figure**5**) associated with the Gastrophyninae and Otophryninae taxa sampled for this study.**

**Node (Figure**5**)**	**Age in mya (95% HPD)**
1. Origin of *Synapturanus*	28.80 (10.36—51.08)
2. Origin of *Otophryne*	28.78 (13.48—47.97)
3. Origin of *Melanophryne* + *Ctenophryne* + *Nelsonophryne* clade	21.85 (11.00—37.36)
4. Origin of *Chiasmocleis*	22.80 (10.42—38.42)
5. Origin of *Chiasmocleis* + *Syncope* clade	37.26 (21.23—54.69)
6. Origin of *Syncope*	13.98 (5.59—25.18)
7. Origin of Gastrophryninae subclade I	64.88 (48.18—78.21)
8. Origin of *Dasypops* + *Myersiella*	22.27 (8.04—37.75)
9. Origin of *Dasypops* + *Stereocyclops* + *Myersiella*	37.83 (23.36—55.53)
10.Origin of *Stereocyclops*	13.94 (5.09—25.92)
11. Origin of Gastrophryninae subclade II*	49.43 (34.29—64.67)
12. Origin of *Arcovomer* + *Hamptophryne* clade	34.50 (21.26—48.89)
13. Origin of *Hamptophryne*	20.86 (8.69-34.99)
14. Origin of Gastrophryninae subclade III*	41.30 (27.91—59.65)
15. Origin of *Elachistocleis*	23.24 (13.30—33.79)
16. Origin of *Elachistocleis* + *Dermatonotus* + *Hypopachus* + *Gastrophryne* clade	33.51 (22.52—46.23)
17. Origin of *Elachistocleis* + *Hypopachus* + *Gastrophryne* clade	30.30 (19.51—41.58)
18. Origin of *Hypopachus*	17.40 (9.94—26.86)
19. Origin of *Hypopachus* + *Gastrophryne*	20.97 (13.09—31.50)
20. Origin of *Gastrophryne*	8.09 (3.49—15.19)

*subclade designations can be found in text.

Our divergence estimates resulted in a tree possessing a mean root height of 82.17 mya (Figure 5). A summary of major node ages (and their respective 95% highest posterior densities [error margins]) is provided in Table 3. Based on the hypothesis that Otophryninae and Gastrophryninae both originated in association with a South American-Antarctica vicariance event in the late Cretaceous [15] and that several *Gastrophryne* species appeared in North America sometime in the Plio-Pleistocene [35], we find that most diversification among gastrophrynine genera occurred during a 30 my period starting in the Eocene and extending into the late to middle Miocene. Interestingly, under the relaxed-clock model, *Otophryne* and *Synapturanus* species appear to have diversified in parallel suggesting that a shared biogeographic event may be responsible for their contemporary diversification during the Oligocene. Our estimates for the origin of *Gastrophryne* and *Hypopachus* at about 21 mya (13.1—31.5 HPD) overlap with previous estimates that estimated the divergence of *Hypopachus* and *Gastrophryne* to be about 17 mya [16].

The inclusion of *Chiasmocleis panamensis* and *Relictivomer pearsei* in *Elachistocleis* restricts the range of *Chiasmocleis* to South America and the northern range of *Elachistocleis* is represented by three species in Panama (*Elachistocleis* sp*., E. panamensis, and E*. *pearsi*). Since the monotypic *Adelastes,* the only NWM genus not sampled in our analyses, is unlikely to be related to *Dermatonotus*, *Elachistocleis*, *Gastrophryne*, or *Hypopachus*, it seems plausible that the North American microhylid radiation is derived from the expansion of a *Dermatonotus*/*Elachistocleis* ancestor. According to our divergence estimates, the node uniting all extant Central and North American microhylids (*Elachistocleis* + *Gastrophryne* + *Hypopachus*) originated in the early Oligocene at about 35 mya. This ancestor could have dispersed from Northern South America during the late Oligocene facilitated by a Central American archipelago connecting these landmasses. A similar pattern of dispersal from the South American Choco region to Central America has been proposed for some dendrobatid lineages during the late Miocene [95]. The presence of a Central American archipelago in the late Miocene [96,97] could explain a much earlier faunal exchange than would be allowed by Plio-Pleistocene land bridges [95].

Although our divergence estimates are broadly consistent with previous hypotheses [44,98], the confidence intervals associated with most estimates are wide (Table 3) and not always consistent with other studies [e.g., 16]. Additionally, given our calibration scheme (two deep secondary, one shallow fossil) and mixed mitochondrial and nuclear sampling it is possible that our divergence estimates may be over [99] or underestimated [100]. As such, we propose this preliminary framework as a hypothesis for gastrophrynine diversification that future investigators will test with a more robust taxonomic and genomic sampling as well as alternative calibration schemes.

### *Morphological diversity:* Gastrophrynines as a study system for developmental plasticity

Using our revised understanding of phylogenetic relationships and divergence estimates within the Gastrophryninae, we see several striking examples of how morphologically variable certain characters have remained over the last ca. 40 my. In particular, two anterior ventral investment bones (i.e., vomers and neopalatines) are recognized as some of the most variable osteological elements in anuran lineages, e. g., either present or absent [1,6,101]. However, except in microhylids, these two elements are not intraspecifically variable in Anura. Gastrophrynine frogs exhibit unusual intraspecific variation in these two elements, e.g., present, absent, reduced, fused, independent. This morphological variation could arise from retained ancestral developmental plasticity in given traits, i.e., plasticity of developmental pathways, to accommodate morphological and ecological constraints of the adult integrated phenotype [102,103]. Environmentally induced variation in development (ontogenetic plasticity) is known to occur in anurans [104-107]. Plasticity in developmental pathways could arise from existing relaxed genetic constraints or ancestral allelic variation in the population [106,108].

Based on recent studies [6,9,11] this study, intraspecific plasticity could have historically misled the diagnoses of anuran systematists who normally treat osteological elements as separate character states when inferring species level relationships when it may be the result of intraspecific plasticity of a given trait. One notable case of this is the *Hamptophryne-Arcovomer* clade where these bones have been reported as: 1) neopalatines: absent in both genera [51,56], reduced in *Hamptophryne*[109], present in *Hamptophryne*[56], polymorphic (present/absent) in *Hamptophryne*[61], present as independent elements in both genera [6] and 2) posterior vomers: reduced in *Hamptophryne* and fused in *Arcovomer*[6,51], reduced in *Hamptophryne*[109], and present in *Arcovomer* and polymorphic (present/absent) in *Hamptophryne*[56,61]. Similar instances of overlapping morphological variation have been reported between *Gastrophryne* and *Hypopachus* see review in [18]. Given this putative plasticity, it seems likely that similar (e.g., level of reduction of a given bony element) or identical character states (e.g. independent loss of a bone or parts of it) in adult morphology have often been interpreted as synapomorphies or autapomorphies when they are actually homoplasies. Thus, future microhylid phylogenetic analyses that aim to incorporate adult morphology should explore and understand the ontogenies of those characters prior to conducting interspecific comparisons and phylogenetic analyses. Understanding the variability of these characters requires detailed developmental studies for which at present there are only three available for Gastrophryninae [6,109,110].

In contrast to the apparent levels of homoplasy in adult morphology, a recent study [111] concluded that Microhyloidea had noticeably lower levels of larval homoplasy than the other major lineages of Neobatrachia. Thus, gastrophrynines may be unique in having low levels of larval homoplasy yet high levels of adult homoplasy. There have been few studies [112] that focus on understanding how the interaction between larval ontogenies and the anuran *Bauplan* relate to the ecological requirements of the adult.

The striking similarity of putative autapomorphic or synapomorphic skeletal traits in phylogenetically divergent lineages within the Gastrophyninae (particularly in the 22 chromosome clade) suggests that these characters may be more appropriately interpreted as homoplastic. While the recurrent nature of this homoplasy could be misdiagnosed or exaggerated by non-standardized documentation, it could also be explained by underlying evolutionary processes like ancestral developmental plasticity. We suspect the latter to be the case given that 1) morphological homoplasy related to ecological specialization has been documented in anurans [7], and references therein, and [2]) characters treated as independent in microhylid systematics studies are often grouped within functional complexes (e.g., cranium, pectoral girdle, pelvic girdle, etc.) that evolve in concert [113,114] and are also likely to be developmentally correlated ([115]; see review of phenotypic integration [103]).

Developmental plasticity is thought to underlie phenotypic plasticity and a populations’ ability to adapt to unstable or changing environments [104-106]. Developmental plasticity of morphological traits, in conjunction with environmental selection, can result in the evolution of new traits [107,108] that trigger speciation or rapid adaptive radiations [116] under variable environmental conditions [107,108,117-120]. A systematist would consider these new traits as potential autapomorphies or synapomorphies to diagnose species and/or to recognize above-species taxonomic categories. In relation to these concepts, the putative instances of morphological homoplasy in closely related gastrophrynines are of particular note, since many lineages have likely diversified not in changing environments but within stable fossorial environments [121]. This scenario has implications for how developmental and phenotypic plasticity of a lineage interact in the *absence* of ecological variability; an underlined prerequisite to studies of phenotypic plasticity [122,123]. In particular, the patterns of diversification we observe in functional complexes, e.g., the anuran palate, may be related to an ancestral developmental plasticity that accommodates the interaction between historical constraints [124] and functional adaptation as lineages diversify within relative stable environmental conditions, e.g. fossoriality. Under the latter scenario, we would expect that lineage diversification would result in evolutionarily independent instances of specialization that produce similar morphological traits, i.e. homoplastic instead of apomorphic traits. Furthermore, this system is of interest since, relative to other vertebrates, anurans have a highly conserved body plan [124], a characteristic that may facilitate a more reliable identification of morphological traits subject to convergence, independent parallelism, or ancestral developmental plasticity. By discussing the interaction between underlying processes and resulting patterns in groups like microhylids, that evolutionary morphologists can make relevant contributions to a research discipline (evo-devo) dominated by studies of developmental and population genetics [102,125].

### Genomic variation in the Gastrophryninae

**Figure 6:**
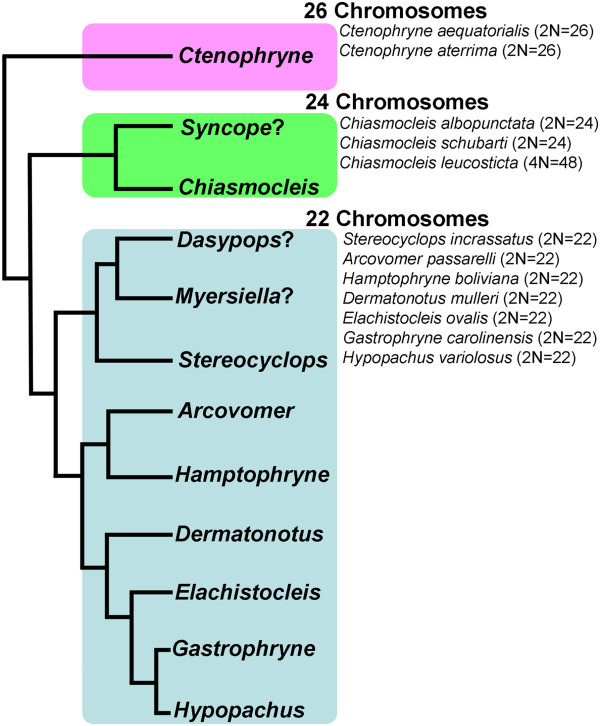
**Chromosome reduction in New World microhylids of the subfamily Gastrophryninae.** Mapping karyotypes on a consensus molecular phylogeny (Figures 2, 3, 4) reveals at least two fusion events may have occurred during the evolution of these frogs. A question mark indicated those genera for which karyotypes are currently unavailable.

Genome structure in microhylids seems to have arisen from a diploid ancestor with 26 chromosomes. This is presumed because the 2N=26 state is present in all of the microhylid subfamilies that have been examined karyologically (Dyscophinae and Cophylinae [126], Otophryninae [127], Gastrophryninae [128], Asterophryinae [129]). There are, however, known deviations from this karyological formula with several subfamilies ranging in chromosome number from 28–22 [128]. One of these instances occurs in the Gastrophryninae where chromosome number ranges from 26–22. By mapping known karyotypes on our Gastrophryninae molecular phylogeny (Figures 2, 3, 4), a putative pattern of chromosome reduction emerges (Figure 6). The earliest detectable branching event in the Gastrophryninae leads to *Ctenophryne* which contains members (*C. aequatorialis* and *C. aterrima*) possessing a 2N=26 karyotype [127]. The next major branching event leads to *Syncope* and *Chiasmocleis* which contains members (*C. albopunctata* and *C. schubarti*) possessing a 2N=24 karyotype. One instance of tetraploidy has been reported for *Chiasmocleis* (*C. leucosticta*), but the 48 chromosomes identified in this species suggest a 24 chromosome ancestral template [130]. The most derived major clade of Gastrophryninae appears to have developed a reduced 22 chromosomes karyotype early in its evolution since *Arcovomer*, *Elachistocleis*, *Gastrophryne*, *Hypopachus*, *Hamptophryne*, *Stereocyclops*, and *Dermatonotus* all possess this condition. The reduction of chromosome number as it relates to morphological character reduction/loss should be explored further.

## Conclusions

Accumulated evidence supports the monophyly of Microhylidae and its major evolutionary lineages. However, relationships among these subfamily lineages remain uncertain. New World microhylids consist of two separate evolutionary lineages, Otophryninae and Gastrophryninae. Otophryninae (2 genera, 5 species) is probably more closely related to old world subfamilies than to Gastrophryninae. Gastrophryninae consists of 12 genera and 66 species (summary in Table 2). Given the levels of phylogenetic diversity observed in our study, it is likely that additional species will be described in the genera *Chiasmocleis*, *Ctenophryne*, *Dermatonotus*, *Elachistocleis*, and *Syncope*. We transfer some species of *Chiasmocleis* to the genera *Syncope* and *Elachistocleis* to render *Chiasmocleis* monophyletic. To better reflect shared evolutionary histories at generic levels, we synonymize *Altigius* with *Hamptophryne*, *Hyophryne* with *Stereocyclops*, and *Nelsonophryne* and *Melanophryne* with *Ctenophryne*. Resolved branches in the Gastrophryninae part of our phylogeny suggest the reduction and loss of morphological and karyological traits. Morphological shifts are mostly related to the reduction or loss of individual elements in functional complexes of the skeleton that may be related to the repeated evolution of a fossorial ecology. Gastrophryninae exhibits a karyological trend towards reduced diploid numbers in the more derived lineages. While most genera have aquatic larvae, there are several reproductive modes that occur in Gastrophryninae including terrestrial development (*Myersiella*) and non-feeding aquatic larvae (*Syncope*). One of the few temperate microhylid radiations (the North American genera *Gastrophryne* and *Hypopachus*) appears to be derived from ancestral stock shared with the South American genus *Elachistocleis*. Our divergence estimates indicate that if Otophryninae and Gastrophryninae originated in the Late Cretaceous, most genus-level diversification occurred during a period spanning the late Oligocene to the Miocene.

## Competing interest

The authors declare that they have no competing interests.

## Authors’ contributions

RdS designed the research; JWS and RdS performed the analyses. RdS, JWS, RS, EG, and SPL collected sequence data. RdS, SR, CFBH did field work to obtain necessary sampling. RdS and JWS wrote the paper with input from RS, EG, MCF, SR, SPL, and CFBH. All authors read and approved the final manuscript.

## Supplementary Material

Additional file 1 Appendix 1. Click here for file

Additional file 2 Appendix 2. Click here for file

## References

[B1] Parker HW A monograph of the frogs of the family Microhylidae 1934 London: British Museum (Natural History)

[B2] Lynch JD Vial JL The transition from archaic to advanced frogs Evolutionary biology of the anurans: contemporary research on major problems 1973 Columbia: University of Missouri Press 133 182

[B3] Blommers-Schlösser RMA Observations on the larval development of some Malagasy frogs, with notes on their ecology and biology (anura: dyscophinae, scaphiophryninae, and cophylinae) Beaufortia 1975 24 7 26

[B4] Wassersug RJ Pyburn WF The biology of the Pe-ret’ toad, Otophryne robusta (Microhylidae), with special consideration of its fossorial larva and systematic relationships Zoological Journal of the Linnean Society 1987 91 137 169 10.1111/j.1096-3642.1987.tb01726.x

[B5] Wu SH Phylogenetic relationships, higher classification, and historical biogeography of the microhyloid frogs (Lissamphibia: Anura: Brevicipitidae and Microhylidae) 1994 University of Michigan Ph. D

[B6] Trueb L Diaz R Blackburn DC Osteology and chondrocranial morphology of Gastrophryne carolinensis (Anura: Microhylidae), with a review of the osteological diversity of New World microhylids Phyllomedusa 2011 10 99 135

[B7] Emerson SB The fossorial frog adaptive zone: a study of convergence and parallelism in the Anura 1971 Los Angeles: University of South California Ph. D

[B8] Haas A Phylogeny of frogs as inferred from primarily larval characters (Amphibia: Anura) Cladistics 2003 19 23 89 10.1111/j.1096-0031.2003.tb00405.x 34905866

[B9] Frost DR Grant T Faivovich J Bain R Haas A Haddad CFB de Sá RO Donnellan SC Raxworthy CJ Wilkinson M Channing A Campbell JA Blotto BL Moler P Drewes RC Nussbaum RA Lynch JD Green D Wheeler WC The amphibian tree of life Bulletin of the American Museum of Natural History 2006 297 1 370

[B10] Pauly GB Hillis DM Cannatella DC Taxonomic freedom and the role of official 778 lists of species names Herpetologica 2009 65 115 128 10.1655/08-031R1.1

[B11] Pyron RA Wiens JJ A large-scale phylogeny of Amphibia with over 2,800 species, and a revised classification of extant frogs, salamanders, and caecilians Mol Phylogenet Evol 2011 61 543 583 10.1016/j.ympev.2011.06.012 21723399

[B12] Frost DR Amphibian species of the world: an online reference 2011 New York, USA: American Museum of Natural History Version 5.5 (accessed on July 1st, 2012) Electronic Database Accessible at http://research.amnh.org/herpetology/amphibia

[B13] Bossuyt F Roelants K Hedges SB, Kumar S Anura The Timetree of Life 2009 New York, U.S.A.: Oxford University Press 357 364

[B14] Frost DR Amphibian species of the world: an online reference 2007 New York, USA: American Museum of Natural History Version 5.0 Electronic Database Accessible at http://research.amnh.org/herpetology/amphibia

[B15] Van Bocxlaer I Roelants K Biju SD Nagaraju J Bossuyt F Late cretaceous vicariance in gondwanan amphibians PLoS One 2006 1 1 74 10.1371/journal.pone.0000074 PMC1762348 17183706

[B16] Van der Meijden A Vences M Hoegg S Boistel R Channing A Myer A Nuclear gene phylogeny of narrow-mouthed toads (Family: Microhylidae) and a discussion of competing hypotheses concerning their biogeographical origins Mol Phylogenet Evol 2007 44 1017 1030 10.1016/j.ympev.2007.02.008 17369057

[B17] Greenbaum E Smith EN de Sá RO Molecular systematics of the middle American genus hypopachus (anura: microhylidae) Mol Phylogenet Evol 2011 61 265 277 10.1016/j.ympev.2011.07.002 21798357 PMC3175309

[B18] Streicher JW Cox CL Campbell JA Smith EN de Sá RO Rapid range expansion in the great plains narrow-mouthed toad (gastrophryne olivacea) and a revised taxonomy for north American microhylids Mol Phylogenet Evol 2012 64 645 653 10.1016/j.ympev.2012.05.020 22659512

[B19] de Sá RO Heyer WR Camargo A A phylogenetic analysis of vanzolinius heyer, 1974 (amphibia, anura, leptodactylidae): taxonomic and life history implications Arquivos do Museu Nacional, Rio de Janeiro 2005 63 707 726

[B20] Streicher JW Crawford AJ Edwards CE Multilocus molecular phylogenetic analysis of the craugastor podiciferus (anura: craugastoridae) species complex in isthmian central America Mol Phylogenet Evol 2009 53 620 630 10.1016/j.ympev.2009.07.011 19602442

[B21] Palumbi S Martin A Romano S McMillan WO Stice L Grabowski G Palumbi S The simple fool’s guide to PCR,v.2.0 1991 Honolulu, HI: Department of Zoology and Kewalo Marine Laboratory. University of Hawaii

[B22] Hillis DM Dixon MT Ribosomal DNA: molecular evolution and phylogenetic inference Q Rev Biol 1991 66 411 453 10.1086/417338 1784710

[B23] Bossuyt F Milinkovitch MC Amphibians as indicators of early Tertiary “out-of-India” dispersal of vertebrates Science 2001 292 93 95 10.1126/science.1058875 11292870

[B24] Liu K Warnow TJ Holder MT Nelesen S Yu J Stamatakis A Linder CR SATe-II: very fast and accurate simultaneous estimation of multiple sequence alignments and phylogenetic trees Syst Biol 2011 61 90 106 22139466 10.1093/sysbio/syr095

[B25] Maddison DR Maddison WP MacClade 4: analysis of phylogeny and character evolution. Version 4.08a 2005 http://macclade.org 10.1159/000156416 2606395

[B26] Miller MA Pfeiffer W Schwartz T Creating the CIPRES science gateway for inference of large phylogenetic trees.Proceedings of the gateway computing environments workshop (GCE) 2010 New Orleans, LA 1 8 14 Nov

[B27] Zwickl DJ Genetic algorithm approaches for the phylogenetic analysis of large biological sequence datasets under the maximum likelihood criterion 2006 Austin: The University of Texas

[B28] Sikes DS Lewis PO PAUPRat: a tool to implement parsi- mony ratchet searches using PAUP* 2001 http://mercury2.iab.uaf.edu/derek_sikes/software2.htm

[B29] Drummond AJ Rambaut A BEAST: Bayesian evolutionary analysis by sampling trees BMC Evol Biol 2007 7 214 10.1186/1471-2148-7-214 17996036 PMC2247476

[B30] Ronquist F Huelsenbeck JP MrBayes version 3.0: Bayesian phylogenetic inference under mixed models Bioinformatics 2003 19 1572 1574 10.1093/bioinformatics/btg180 12912839

[B31] Tamura K Peterson D Peterson N Stecher G Nei M Kumar S MEGA5: molecular evolutionary genetics analysis using maximum likelihood, evolutionary distance, and maximum parsimony methods Mol Biol Evol 2011 28 2731 2739 10.1093/molbev/msr121 21546353 PMC3203626

[B32] Kuhner MK Felsenstein J A simulation comparison of phylogeny algorithms under equal and unequal evolutionary rates Mol Biol Evol 1994 11 459 468 8015439 10.1093/oxfordjournals.molbev.a040126

[B33] Wilgenbusch JC Warren DL Swofford DL System for graphical exploration of MCMC convergence in Bayesian phylogenetic inference 2004 Tallahassee, USA: FloridaState University 10.1093/bioinformatics/btm388 17766271

[B34] Figtree v 1.5 Available from http://tree.bio.ed.ac.uk/software/figtree

[B35] Sanchiz B Encyclopedia of paleoherpetology. Part 4. Salientia 1998 München: Pfeil 275

[B36] Thorne JL Kishino H Divergence time and evolutionary rate estimation with multilocus data Syst Biol 2002 51 689 702 10.1080/10635150290102456 12396584

[B37] Sanderson MJ Estimating absolute rates of molecular evolution and divergence times: a penalized likelihood approach Mol Biol Evol 2002 19 101 109 10.1093/oxfordjournals.molbev.a003974 11752195

[B38] Holman JA Farlow JO Fossil frogs and toads of North America Indiana university press 2003 Bloomington: Indiana 246

[B39] Gernhard T The conditioned reconstructed process J Theor Biol 2008 253 769 778 10.1016/j.jtbi.2008.04.005 18538793

[B40] Rambaut A Drummond AJ Tracer v1.4 2007 Available from http://beast.bio.ed.ac.uk/Tracer

[B41] Maddison WP Missing data versus missing characters in phylogenetic analysis Syst Biol 1993 42 576 581 10.1093/sysbio/42.4.576

[B42] Wiens JJ Morrill MC Missing data in phylogenetic analysis: reconciling results from simulations and empirical data Syst Biol 2011 60 719 731 10.1093/sysbio/syr025 21447483

[B43] Hillis DM Bull JJ An empirical test of bootstrapping as a method for assessing confidence in phylogenetic analysis Syst Biol 1993 42 182 192

[B44] Kurabayashi A Matsui M Belabut DM Yong HS Ahmad N Sudin A Kuramoto M Hamidy A Sumida M From Antarctica or Asia? New colonization scenario for Australina-New Guinean narrow mouth toads suggested from the finding on a mysterious genus Gastrophrynoides BMC Evol Biol 2011 11 175 10.1186/1471-2148-11-175 21689462 PMC3141433

[B45] Ford L Cannatella C The major clades of frogs Herpetological Monographs 1993 7 94 117

[B46] Matsui M Hamidy A Belabut D Ahmad N Panha S Sudin A Khonsue W Oh H-S Yong H-S Jiang J-P Nishikawa K Systematic relationships of oriental tiny frogs of the family microhylidae (amphibia, anura) as revealed by mtDNA genealogy Mol Phylogenet Evol 2011 61 167 176 10.1016/j.ympev.2011.05.015 21658458

[B47] Köhler F Günther R The radiation of microhylid frogs (Amphibia: Anura) on New Guinea: A mitochondrial phylogeny reveals parallel evolution of morphological and life history traits and disproves the current morphology-based classification Mol Phylogenet Evol 2008 47 353 365 10.1016/j.ympev.2007.11.032 18249011

[B48] Günther R Metamagnusia and Pseudocallulops, two new genera of microhylid frogs from New Guinea (Amphibia, Anura, Microhylidae) Zoosystematics and Evolution, Mitteilungen aus dem Museum für Naturkunde in Berlin 2009 85 171 187

[B49] Andreone F Vences M Vieites DR Glaw F Meyer A Recurrent ecological adaptations revealed through a molecular amalysis of the secretivce cophyline frogs of Madagascar Mol Phylogenet Evol 2005 34 315 322 10.1016/j.ympev.2004.10.013 15619444

[B50] Dunn ER Notes on South American frogs of the family Microhylidae American Museum Museum of Natural History, Novitates 1949 1419 1 21

[B51] Carvalho AL A preliminary synopsis of the genera of American microhylid frogs Occasional Papers of the Museum of Zoology, University of Michigan 1954 555 1 19

[B52] Lehr E Trueb L Diversity among New World microhylid frogs (Anura: Microhylidae): morphological and osteological comparisons between Nelsonophryne (Günther 1901) and a new genus from Peru Zoological Journal of the Linnean Society 2007 149 583 609 10.1111/j.1096-3642.2007.00270.x

[B53] Donnelly MA de Sá RO Guyer C Description of the tadpoles of Gastrophryne pictiventris and Nelsonophryne aterrima (Anura: Microhylidae), with a review of morphological variation in free-swimming microhylid larvae American Museum Novitates 1990 2976 1 19

[B54] Lehr E Trueb L Venegas PJ Arbelaez E Descriptions of the tadpoles of two Neotropical microhylid frogs, Melanophryne carpish and Nelsonophryne aequatorialis (Anura: Microhylidae) J Herpetol 2007 41 581 589 10.1670/06-252.1

[B55] Schlüter A Salas AW Reproduction, tadpoles, and ecological aspects of three syntopic microhylid species from Peru (Amphibian: Microhylidae) Stuttgarter Beiträge zur Naturkunde 1991 458 1 17

[B56] Zweifel RG A new genus and species of microhylid frog from the Cerro de la Neblina region of Venezuela and a discussion of relationships among new world microhylid genera American Museum Novitates 1986 2863 1 24

[B57] Caramaschi U Cruz CAG A new species of Chiasmocleis Méhely,1904 from Brazilian Amazonia (Amphibia, Anura, Microhylidae) Boletim do Museu Nacional (N.S.) Zoologia 2001 469 1 8

[B58] Moravec J Köhler J A new species of Chiasmocleis (Anura: Microhylidae) from the Iquitos region, Amazonian Peru, with possible direct development Zootaxa 2007 1605 59 67

[B59] Duellman WE Trueb L Biology of amphibia 1986 New York: McGraw-Hill

[B60] Krügel P Richter S Syncope antenori—a bromeliad breeding frog with free-swimming, nonfeeding tadpoles (Anura, Microhylidae) Copeia 1995 955 963

[B61] Forlani M Morfologia do gênero Chiasmocleis Méhely, 1904 (Anura, Microhylidae, Gastrophryninae), e suas implicações filogenéticas. MS. Sc. Dissertation 2010 Instituto de Biociências da Universidade de São Paulo 138

[B62] Nascimento FAC Skuk GO O girino de Chiasmocleis alagoanus Cruz, Caramaschi & Freire, 1999 (Anura: Microhylidae) Biota Neotropica 2006 6 3

[B63] Oliveira Filho JC Giaretta AA Tadpole and advertisement call of Chiasmocleis albopunctata (Anura, Microhylidae) from Brazil Zootaxa 2006 1353 63 68

[B64] Duellman EE The biology of an equatorial herpetofauna in Amazonian Ecuador Miscellaneous Publications Museum of Natural History, University of Kansas 1978 65 1 352

[B65] Wogel H Abrunhosa PA Prado GM The tadpole of Chiasmocleis carvalhoi and the advertisement calls of three species of Chiasmocleis (Anura, Microhylidae) from the Atlantic rainforest of southeastern Brazil Phyllomedusa 2004 3 133 140

[B66] Langone JA Lavilla EO Echeverría DD Mangione S Segalla MV Morfologia externa e interna de la larva de Chiasmocleis leucosticta (Boulenger, 1888) (Amphibia, Anura, Microhylidae) Publicaciónn extra del Museo Nacional de Historia Natural y Antropologia 2007 2 1 17

[B67] Santana DJ Motta AP Pirani RM Silva ET Feio RN Advertisement call and tadpole of chiasmocleis mantiqueira (anura, microhylidae) J Herpetol 2012 46 14 18 10.1670/10-104

[B68] Hero JM An illustrated key to tadpoles occurring in the Central Amazon rainforest, Manaus, Amazonas, Brasil. Amazoniana 1990 11 201 262

[B69] Rodrigues DJ Menin M Lima AP Mokrss KS Tadpole and vocalizations ofChiasmocleis hudsoni(Anura, Microhylidae) in Central Amazonia, Brazil. Zootaxa 2008 1680 55 58

[B70] Dunn ER Trapido H Evans H A new species of the microhylid frog genus Chiasmocleis from Panama American Museum Museum of Natural History, Novitates 1948 1376 1 8

[B71] Tarigo Rocha M Redescrição e osteologia de Hyophryne histrio Carvalho, 1954 e sua posição filogenética em Gastrophryninae (Amphibia, Anura, Microhylidae) 2009 MS. Sc. Dissertation, Museu Nacional, Universidade Federal do Rio de Janeiro 147

[B72] Wild ER New genus and species of Amazonian microhylid frog with a phylogenetic analysis of new world genera Copeia 1995 837 849

[B73] Griffiths I Carvalho AL On the validity of employing larval characters as major phyletic indices in Amphibia, Salientia Rev Bras Biol 1965 25 113 121

[B74] Wogel H Abrunhosa PA Pombal JP Jr Girinos de cinco espécies de anuros do sudeste do Brasil (Amphibia: Hylidae, Leptodactylidae, Microhylidae) Boletim do Museu Nacional, (N. S.) Zoologia 2000 427 1 16

[B75] Nelson CE Lescure J The taxonomy and distribution of Myersiella and Synapturanus (Anura: Microhylidae) Herpetologica 1975 31 389 397

[B76] Nelson CE Mating calls of the Microhylinae: descriptions and phylogenetic and ecological considerations Herpetologica 1973 29 163 176

[B77] Cruz CAG Peixoto OL Notas sobre o girino de Dasypops schrichi Miranda-Ribeiro (Amphiba, Anura, Microhylidae) Rev Bras Biol 1978 38 297 299

[B78] Izecksohn E Jim J Albuquerque SL Mendonça WF Observações sobre o desenvolvimento e os hábitos de Myersiella subnigra (Miranda-Ribeiro) (Amphibia, Anura, Microhylidae) Arquivos do Museu Nacional 1971 24 69 73

[B79] Menin M Rodrigues DJ Lima AP Clutches, tadpoles and advertisement calls of Synapturanus mirandaribeiroi and S. cf. salseri in Central Amazonia, Brazil Herpetological Journal 2007 17 86 91

[B80] Nelson CE Systematic studies of the North American microhylid genus Gastrophryne J Herpetol 1972 6 111 137 10.2307/1562801

[B81] Cei JM Amphibians of Argentina Monitore Zoologico Italiano, N. S. Monographia 1981 2 1 609

[B82] Lavilla EO The tadpole of Dermatonotus muelleri (Anura: Microhylidae) Bolletino Museum Regionale de Science Naturale Torino 1992 10 63 71

[B83] Vizotto LD Desenvolvimento de Anuros de região norte-ocidental do Estado de São Paulo Rec Fac Filos Cienc Lets SJ Rio Preto Zool Especial 1967 1 161

[B84] Williams JD Gudynas E Descripcion de la larva de Elachistocleis bicolor (Valenciennes, 1838) (Anura:Microhylidae) Amphibia-Reptilia 1987 8 225 229 10.1163/156853887X00261

[B85] Vera Candioti MF Morfología larval de Chiasmocleis panamensis, con comentarios sobre la variabilidad morfológica interna en renacuajos de Microhylidae (Anura) Alytes 2006 24 91 108

[B86] Lynch JD The tadpoles of frogs and toads found in the lowlands of northern Colombia Revista de la Academia Colombiana de Ciencias 2006 30 443 457

[B87] Kenny JS The Amphibia of Trinidad Studies of the Fauna of the Curaçao Caribbean Island 1969 29 1 78

[B88] Orton GL The unknown tadpole Turtox News 1946 24 131 132

[B89] Altig R A key to the tadpoles of the continental United States and Canada Herpetologica 1970 26 180 207

[B90] Nelson CE Altig R Tadpoles of the microhylids Gastrophryne elegans and G. usta Herpetologica 1972 28 381 383

[B91] Stuart LC Another new Hypopachus from Guatemala Proceedings of the Biological Society of Washington 1941 54 125 128

[B92] Stuart LC Comments on the herpetofauna of the Sierra de Los Cuchumatanes of Guatemala Occasional Papers of the Museum of Zoology, University of Michigan 1943 471 1 28

[B93] Taylor EH Tadpoles of Mexican Anura University of Kansas Science Bulletin 1942 28 37 55

[B94] Savage JM Vial JL The geographic distribution of frogs: patterns and predictions The Evolutionary Biology of the Anurans 1973 13 Columbia, Missouri: Univ. of Missouri Press 351 454

[B95] Santos JC Coloma LA Summers K Caldwell JP Ree R Cannatella DC Amazonian Amphibian Diversity Is Primarily Derived from Late Miocene Andean Lineages PLoS Biol 2009 7 448 461 10.1371/journal.pbio.1000056 PMC2653552 19278298

[B96] Coates AG Obando JA Jackson JBC, Budd AF, Coates AG The geological evolution of the Central American Isthmus Evolution and environment in tropical America 1996 Chicago, Illinois: The University of Chicago Press 21 56

[B97] Coates AG Collins LS Aubry MP Berggren WA The geology of the Darien, Panama, and the late Miocene-Pliocene collision of the Panama arc with northwestern South America The Geological Society of America Bulletin 2004 116 1327 1344 10.1130/B25275.1

[B98] Roelants K Gower DJ Wilkinson M Loader SP Biju SD Guillaume K Moriau L Bossuyt F Global patterns of diversification in the history of modern amphibians Proc Natl Acad Sci 2007 104 887 892 10.1073/pnas.0608378104 17213318 PMC1783409

[B99] Ho SYW Phillips MJ Cooper A Drummond AJ Time dependency of molecular rate estimates and systematic overestimation of recent divergence times Mol Biol Evol 2005 22 1561 1568 10.1093/molbev/msi145 15814826

[B100] Near TJ Eytan IR Dornburg A Kuhn KL Moore D Wainwright PC Friedman M Smith WL Resolution of ray-finned fish phylogeny and timing of diversification Proc Natl Acad Sci 2012 109 13698 13703 10.1073/pnas.1206625109 22869754 PMC3427055

[B101] Trueb L Vial JL Bones, frogs, and evolution Evolutionary biology of Anurans: contemporary research on major problems 1973 Columbia: University of Missouri Press 65 132

[B102] Wake DB Wake MH Specht CD Homoplasy: From detecting pattern to determine process and mechanism of evolution Science 2011 331 1032 1035 10.1126/science.1188545 21350170

[B103] Murren CJ The integrated phenotype Integrative and Comparative Biology 2012 52 64 76 10.1093/icb/ics043 22593559

[B104] Pfenning DW The adaptive significance of an environmentally-cued developmental switch in an anuran tadpole Oecologia 1990 85 101 107 10.1007/BF00317349 28310961

[B105] Gomez-Mestre I Buchholz DR Developmental plasticity mirrors differences among taxa in spadefoot toads linking plasticity and diversity Proceedings of the National Academy of Sciences, USA 2006 103 19021 19026 10.1073/pnas.0603562103 PMC1748170 17135355

[B106] Leichty AR Pfenning DW Jones CD Pfenning KS Relaxed genetic constraint is ancestral to the evolution of phenotypic plasticity Integrative and Comparative Biology 2012 52 16 30 10.1093/icb/ics049 22526866 PMC3381942

[B107] Pfenning DW Murphy PJ How fluctuating competition and phenotypic plasticity mediate species divergence Evolution 2002 56 1217 28 12144021 10.1111/j.0014-3820.2002.tb01433.x

[B108] Wund M Baker JA Clancy B Golub J Foster SA A test of the ‘flexible stem’ model of evolution: ancestral plasticity, genetic accommodation, and morphological divergence in the threespine stickleback radiation Am Nat 2008 172 449 62 10.1086/590966 18729721

[B109] de Sá RO Trueb L Osteology, skeletal development, and chondrocranial structure of Hamptophryne boliviana (Anura: Microhylidae) J Morphol 1991 209 311 330 10.1002/jmor.1052090307 29865541

[B110] Fabrezi MS Quinzio S Goldberg J de Sá RO The Development of Dermatonotus muelleri (Anura: Microhylidae: Gastrophyninae) J Herpetol 2012 46 363 380 10.1670/11-194

[B111] Roelants K Haas A Bossuyt F Anuran radiations and the evolution of tadpole morphospace Proc Natl Acad Sci USA 2011 108 8731 8736 10.1073/pnas.1100633108 21555583 PMC3102353

[B112] Hanken J Model systems versus outgroups: alternative approaches to the study of head development and evolution Am Zool 1993 33 448 456

[B113] Emerson SB The ilio-sacral articulation in frogs: form and function Biol J Linn Soc 1979 11 153 168 10.1111/j.1095-8312.1979.tb00032.x

[B114] Emerson SB Frog postcranial morphology: Identification of a functional complex Copeia 1982 3 603 613

[B115] Emerson SB Morphological variation in frog pectoral girdle: testing alternatives to a traditional adaptive explanation Evolution 1984 38 376 388 10.2307/2408496 28555922

[B116] Pfenning DW Wund MA Snell-Rood EC Cruickshank T Schlichting CD Moczek AP Phenotypic plasticity’s impacts on diversification and speciation Trends Ecol Evol 2010 25 459 467 10.1016/j.tree.2010.05.006 20557976

[B117] West-Eberhard MJ Phenotypic plasticity and the origin of diversity Annual Review of Ecology and Systematics 1989 20 249 78 10.1146/annurev.es.20.110189.001341

[B118] West-Eberhard MJ Developmental plasticity and the origin of species differences Proceedings of the National Academy of Sciences, USA 2005 102 6543 49 10.1073/pnas.0501844102 PMC1131862 15851679

[B119] Schlichting CD DeWitt TJ, Scheiner SM The role of phenotypic plasticity in diversification Phenotypic Plasticity: functional and conceptual approaches 2004 New York: Oxford University Press 191 200

[B120] Whitman DW Agrawal AA Whitman DW, Ananthakrishnan TN What is phenotypic plasticity and why is it important? Phenotypic Plasticity of Insects 2009 Science Publishers 1 63

[B121] Vitt LJ Caldwell JP Herpetology 2009 3 Academic 698

[B122] Pigliucci M Phenotypic Plasticity: Beyond Nature and Nurture 2001 Baltimore, MD: Johns Hopkins University Press

[B123] Nijhout HF German RZ Developmental causes of allometry: New models and implications for phenotypic plasticity and evolution Integrative and Comparative Biology 2012 52 43 52 10.1093/icb/ics068 22634387 PMC6366551

[B124] Handrigan GR Wassersug RJ The anuran Bauplan: a review of the adaptive, developmental, and genetic underpinnings of frog and tadpole morphology Biol Rev 2007 82 1 25 17313522 10.1111/j.1469-185X.2006.00001.x

[B125] Love AC Evolutionary morphology, innovation, and the synthesis of evolutionary and developmental biology Biology and Philosophy 2003 18 309 345 10.1023/A:1023940220348

[B126] Blommers-Schlösser RMA Chromosomal analysis of twelve species of Microhylidae (Anura) from Madagascar Genetica 1976 46 199 210 10.1007/BF00121036

[B127] Kuramoto M A list of chromosome numbers of anuran amphibians Bulletin Fukuoka University Education 1990 39 83 127

[B128] Bogart JP Nelson CE Evolutionary implications from karyotypic analysis of frogs of the families Microhylidae and Rhinophrynidae Herpetologica 1976 32 199 208

[B129] Mahony M Donnellan SC Alpine K Karyotypes of Australo-Papuan microhylid frogs (Anura: Microhylidae) Herpetologica 1992 48 184 192

[B130] Kasahara S Haddad CFB Karytotypes of two Brazilian microhylid frogs of the genus Chiasmocleis, including a new case of polyploidy J Herpetol 1997 31 139 142 10.2307/1565345

