# Supplementary material for: Molecular phylogeny of microhylid frogs (Anura: Microhylidae) with emphasis on relationships among New World genera
Source: BMC Evol Biol. 2012 Dec 10;12:241. doi: 10.1186/1471-2148-12-241 (PMC3561245; doi:10.1186/1471-2148-12-241)
Supplement: Additional file 1 — Appendix 1. [file 1471-2148-12-241-S1.doc]

| **SPECIMEN** | **NUMBER** | **LOCALITY** | **GENBANK ACCESION NUMBERS** | |  |  |
| --- | --- | --- | --- | --- | --- | --- |
| **OUTGROUPS** |  |  | **16S** | **TYR** | **BDNF** | **28S** |
| *Gephyromantis esculpturatus* | 2002 0187 | Ranomafana NP, Madagascar | KC180055 | None | None | KC179828 |
| *Ptychadena anchietae* | RdS 905 | Dar Es Salaam University, Dar Es Salaam, Tanzania | KC179964 | KC180299 | KC180108 | KC179935 |
| *Amietia angolensis* | RdS 871 | Mumba Village, Tanzania | KC179968 | KC180297 | KC180104 | KC179832 |
| *Strongylopus fuelleborni* | RdS 994 | Sáo Hill Swamp (1.5 hours from Iringa), Tanzania | None | KC180300 | None | KC179951 |
| *Tomopterna tuberculosa* | RdS 880 | Tatanda Village, Tanzania | KC179967 | KC180298 | KC180107 | KC179959 |
| *Lithobates* sp. | RdS 541 | Cockscom Basin, Stann Creek District, Belize | KC180010 | KC180292 | KC180082 | KC179938 |
| *Polypedates leucomystax* | K3031 | Doi Chiang Dao, Thailand | KC180033 | KC180269 | KC180096 | KC179932 |
| *Polypedates megacephalus* | K3221 | Ban Sop Choun, Laos | KC180075 | KC180271 | KC180112 | KC179933 |
| *Arthroleptis xenodactyloides* | RdS 864 | Tegetero Village, Ulugurus Mts, Tanzania | KC180024 | KC180296 | KC180101 | KC179838 |
| *Arthroleptis tanneri* | RdS 929 | West Usumbaras Mts, Mazumbai, Tanzania | DQ283427 | DQ283020 | KC180092 | DQ283736 |
| *Kassina senegalensis* | RdS 803 | Kibebe Farm, Iringa, Tanzania | DQ283437 | DQ283026 | None | DQ283746 |
| *Afrixalus ulugurensis* | RdS 845 | Tegetero Village, Ulugurus Mts, Tanzania | None | KC180294 | None | KC179831 |
| *Hyperolius spinigularis* | RdS 835 | Tegetero Village, Ulugurus Mts, Tanzania | KC179966 | KC180293 | None | KC179891 |
| *Hemisus sudanensis* | T471 | Comoe, NP, Ivory Coast | KC180076 | KC180308 | None | KC179889 |
| *Hemisus marmoratus* | RdS 916 | Masai Camp, Arusha, Tanzania | AY843594 | DQ283022 | KC180102 | DQ283738 |
| *Breviceps mossambicus* | RdS 903 | Morogoro, Tanzania | DQ283155 | DQ283013 | KC180103 | DQ283546 |
| *Callulina kisiwamsitu* | RdS 936 | Mazumbai, West Usumbaras Mts, Tanzania | DQ283429 | DQ283021 | KC180213 | DQ283737 |
| *Spelaeophryne methneri* | MW 1850 (NHM Field Series) | Tanzania | KC179989 | KC180282 | KC180093 | KC179946 |
| *Probreviceps macrodactylus* | RdS 863 | Tegetero Village, Ulugurus Mts, Tanzania | None | KC180295 | KC180081 | KC179934 |
| **HOPLOPHRYNINAE** |  |  |  |  |  |  |
| *Hoplophryne rogersi* | RdS 949 | East Usumbaras Mts, Adjacent to Amanai Nature Reserve, Tanzania | DQ283419 | DQ283015 | KC180215 | DQ283730 |
| **SCAPHIOPHRYNINAE** |  |  |  |  |  |  |
| *Scaphiophryne calcarata* | ZSM 115/2002 | Pic St. Louis, Tolagnaro, Madagascar | KC180054 | KC180319 | KC180097 | KC179830 |
| *Scaphiophryne madagascariensis* | pet trade | No locality data | KC180047 | KC180305 | KC180158 | KC179944 |
| *Scaphiophryne madagascariensis* | FGMV 2002.2120 | Ankaratra, Manjakatompo, Madagascar | KC180053 | None | KC180089 | KC179829 |
| **DYSCOPHINAE** |  |  |  |  |  |  |
| *Dyscophus antongilii* | ZSM 1053/2003 | Maroantsetra, Madagascar | KC180056 | KC180320 | KC180090 | KC179872 |
| *Dyscophus guineti* | pet trade | No locality data | DQ283434 | DQ282999 | KC180161 | DQ283743 |
| **MICROHYLINAE** |  |  |  |  |  |  |
| *Micryletta inornata* | AK 1090 | Bab Tung Tao, Surat Thani, Bab Na San, Thailand | None | KC180233 | None | KC179914 |
| *Micryletta inornata* | K 3246 | Ban Sop Chuna, Luangprabang Province, Laos | KC180027 | KC180272 | KC180131 | KC179918 |
| *Micryletta inornata* | K1956 | Long Nai Kao, Phongsaly Province, Laos | KC180067 | KC180268 | KC180209 | KC179916 |
| *Micryletta inornata* | K 3068 | No locality data | None | KC180270 | KC180114 | KC179917 |
| *Micryletta inornata* | FMNH 255121 | Boulapha, Khammouan, Laos | KC179997 | None | KC180117 | KC179915 |
| *Uperodon* | T1 | No locality data | KC180072 | KC180309 | None | KC179960 |
| *Ramanella variegata* | 0019C | No locality data | KC180007 | KC180217 | KC180128 | KC179937 |
| *Ramanella obscura* | MM 5980 | Sri Lanka | KC180006 | None | KC180142 | KC179936 |
| *Metaphrynella sundana* | FMNH 231203 | Lahad Datu, Sabah, Malaysia | KC179996 | None | KC180091 | None |
| *Kaloula baleata* | ROM 32932 | No locality data | KC179969 | None | KC180145 | KC179904 |
| *Kaloula baleata* | ROM 32925 | No locality data | KC180032 | None | KC180133 | None |
| *Kaloula picta* | USFS 56931 | No locality data | KC180019 | KC180315 | KC180137 | KC179905 |
| *Kaloula pulchra* | RdS 1200 | No locality data | None | KC180289 | KC180204 | KC179940 |
| *Kaluola pulchra* | USFS 34083 | Sagaing, Chattgub WS, Myanmar | KC180025 | KC180314 | KC180100 | KC179906 |
| *Chaperina fusca* | RMB 3031 | Philippines | KC180012 | None | KC180106 | KC179843 |
| *Chaperina fusca* | RMB 3053 | Philippines | KC180003 | None | None | KC179844 |
| *Microhyla achatina* | RMB 2620 | Indonesia | KC180049 | None | KC180095 | KC179909 |
| *Glyphoglossus molossus* | USFS 34043 | Sagaing, Chattgub WS, Myanmar | KC180052 | KC180310 | KC180186 | KC179883 |
| *Glyphoglossus molossus* | USFS 34044 | Sagaing, Chattgub WS, Myanmar | KC180000 | KC180313 | KC180216 | KC179884 |
| *Microhyla butleri* | CAS210751 | Hlawgaw Wildlife Park, Yangon, Myanmar | KC180042 | None | None | KC179911 |
| *Calluela yunnanensis* | FMNH 232988 | Sichuan, China | KC179992 | None | None | KC179840 |
| *Microhyla berdmorei* | CAS204876 | Alaungdaw Kathapa National Park, Sagaing, Myanmar | KC179981 | None | KC180094 | KC179910 |
| *Microhyla heymonsi* | CAS210748 | Hlawgaw Wildlife Park, Yangon, Myanmar | KC179993 | KC180219 | None | KC179912 |
| *Microhyla ornata* | CAS230957 | Ma Gawe Reserve, Kalaw, Taunggyi, Sha, Myanmar | KC179995 | KC180221 | KC180087 | KC179913 |
| **ASTEROPHRYINAE** |  |  |  |  |  |  |
| *Oreophryne* sp. | 2289 | LagifuRidge, SHP, Papua New Guinea | KC180073 | None | KC180088 | KC179926 |
| *Oreophryne anulata* | H1366 | No locality data | KC180016 | None | KC180199 | KC179925 |
| *Oreophryne* sp. | 9731 | Porgera, Enga, Papua New Guinea | KC180009 | None | KC180200 | KC179924 |
| *Cophixalus* sp. | CCA476L | Bakia Village, Garaina, Moroboe, Papua New Guinea | KC180051 | KC180244 | KC180170 | KC179864 |
| *Cophixalus* sp. | 7741 | Salawati Island, Indonesia | KC180058 | None | KC180173 | KC179863 |
| *Copiula* sp. | 3235 | Libano, SHP, Papua New Guinea | KC180066 | KC180224 | KC180136 | KC179866 |
| *Copiula oxyrhina* | 2721 | Misima, Milne Bay, Papua New Guinea | KC179970 | None | None | KC179865 |
| *Austrochaperina derongo* | 2293 | Moro, SHP, Papua New Guinea | None | KC180220 | KC180177 | KC179839 |
| *Liophryne schlaginhoufeni* | 2380 | Moro, SHP, Papua New Guinea | KC180043 | None | KC180169 | KC179907 |
| *Sphenophryne* sp. | 2541 | Kopi Camp, Enga, Papua New Guinea | KC180035 | KC180222 | KC180174 | KC179947 |
| *Callulops robustus* | 45452 | Namosado, Papua New Guinea | KC179971 | KC180228 | KC180184 | KC179841 |
| *Cophixalus* sp. | CCA series | Papua New Guinea | KC180050 | KC180245 | KC180187 | KC179945 |
| *Metamagnusia slateri* | 2099 | Gobe, SHP, Papua New Guinea | KC179974 | KC180218 | KC180183 | KC179842 |
| *Xenorhina obesa* | 6139 | Foyas, Indonesia | KC180002 | KC180230 | KC180134 | KC179962 |
| *Xenorhina* sp. | 3137 | Darai, Gulf, Papua New Guinea | KC180015 | KC180223 | KC180188 | KC179961 |
| *Xenorhina* sp. | 3689 | Utai SP, Papua New Guinea | KC180013 | KC180225 | KC180148 | KC179963 |
| **PHRYNOMERINAE** |  |  |  |  |  |  |
| *Phrynomantis microps* | RdS 1196 | Pet trade | KC179965 | KC180288 | KC180208 | KC179939 |
| **KALOPHRYNINAE** |  |  |  |  |  |  |
| *Kalophrynus interlineatus* | USFS 34285 | Sagaing, Chattgub WS, Myanmar | KC180004 | KC180311 | KC180203 | KC179902 |
| *Kalophrynus pleurostigma* | AK 1097 | NSB, Ratchathani, Thailand | KC180011 | None | KC180147 | KC179903 |
| **OTOPHRYNINAE** |  |  |  |  |  |  |
| *Synapturanus mirandaribeiroi* | SMNS 12078 | Iwokrama Forest, Guyana | KC180018 | None | KC180086 | KC179953 |
| *Synapturanus salseri* | 55 | Ceguera, Cerro Autana, Amazonas, Venezuela | None | KC180229 | KC180113 | KC179954 |
| *Synapturanus* sp. | MW 1004 (NHM Field Series) | Iwokrama Forest, Guyana | KC180029 | KC180283 | KC180123 | KC179952 |
| *Otophryne steyermarki* | ROM 39677 | Mount Ayanganna, Guyana | KC180078 | KC180301 | KC180155 | KC179928 |
| *Otophryne robusta* | ROM 39679 | Mount Ayanganna, Guyana | KC180034 | KC180302 | KC180127 | KC179927 |
| **COPHYLINAE** |  |  |  |  |  |  |
| *Rhombophryne testudo* | ZSM 474/2000 | Nosy Be (Lokobe), Madagascar | KC180070 | None | KC180083 | KC179943 |
| *Rhombophryne testudo* | T2 | Madagascar | None | EF395990 | EF396031 | None |
| *Rhombophryne alluaudi* | T3 | Madagascar | None | EF395988 | EF396028 | None |
| *Rhombophryne alluaudi* | ZSM 3/2002 | Andasibe, Madagascar | None | None | KC180098 | KC179930 |
| *Anodonthyla montana* | MV 2001.530 | Andringitra (Cuvette Boby), Madagascar | None | None | KC180084 | KC179835 |
| *Plethodontohyla* sp. | ZSM 649/2003 | Ranomafana, Madagascar | None | None | KC180085 | KC179931 |
| *Platypelis grandis* | ZSM 652/2003 | Ranomafana, Madagascar | KC180057 | KC180321 | None | KC179929 |
| **GASTROPHRYNINAE** |  |  |  |  |  |  |
| *Melanophryne barbatula* |  | Parque Nacional Yanachaga-Chemillen, Oxapampa,Pasco, Peru | KC179994 | KC180260 | KC180205 | KC179908 |
| *Nelsonophryne aterrima* | QCAZ 17124 | Alto Tambo, Esmerladas, Ecuador | KC180028 | KC180285 | KC180175 | KC179923 |
| *Nelsonophryne aequatorialis* | QCAZ 31243 | Cuenca, Azuay, Ecuador | KC180017 | KC180287 | KC180109 | KC179922 |
| *Ctenophryne geayi* | WED 56812 | S slope Abra Barro Negro, Chachapoya: Amazonas: Peru | KC179998 | None | KC180162 | KC179868 |
| *Ctenophryne geayi* | ROM 40138 | Reserva Tambopata, Madre de Dios, Peru | KC180020 | KC180303 | KC180190 | KC179867 |
| *Syncope carvalhoi* | KU 215720 | San Juan de Pacaysapa, San Martin, Peru | KC179972 | KC180275 | KC180119 | KC179958 |
| *Syncope antenori* | QCAZ 23824 | Yasuni, Orellanad, Ecuador | None | KC180286 | None | KC179957 |
| *Syncope* sp. | 31 | Villa Anguilla at Rio Nanay, Loreto, Peru | KC180065 | KC180306 | KC180116 | KC179955 |
| *Syncope* sp. | 63 | Villa Anguilla at Rio Nanay, Loreto, Peru | KC180074 | KC180307 | KC180110 | KC179956 |
| *Chiasmocleis magnova* | 7 | 31 KM SW of Iquitos, Loreto, Peru | KC180062 | KC180248 | KC180130 | KC179856 |
| *Chiasmocleis bassleri* | NMPGV 71148 | Puerto Almendras, 17 KM SW of Iquitos, Loreto, Peru | None | KC180284 | KC180171 | None |
| *Chiasmocleis hudsoni* | MAD 116 | Muri Scrub Camp, Iwokrama Forest, Guyana. | KC180060 | KC180280 | KC180099 | None |
| *Chiasmocleis shudikarensis* | JIW 458 | Lely Mts., Sipaliwini District, Suriname. | KC180008 | KC180266 | KC180121 | KC179860 |
| *Chiasmocleis ventrimaculata* | ROM 40139 | Reserva Tambopata, Madre de Dios, Peru | KC180041 | KC180304 | KC180143 | KC179862 |
| *Chiasmocleis ventrimaculata* | KU 215540 | Puerto Maldonado, Cuzco Amazonico, Madre de Dios, Peru | KC180059 | KC180274 | KC180172 | KC179861 |
| *Chiasmocleis leucosticta* | T5 | Brazil | KC180039 | KC180243 | KC180167 | KC179855 |
| *Chiasmocleis albopunctata* | JMP 218 | Bolivia | KC179990 | KC180267 | KC180149 | KC179847 |
| *Chiasmocleis albopunctata* | C621 | Mata São José, Rio Claro, SP, Brazil | KC179991 | KC180240 | KC180139 | KC179850 |
| *Chiasmocleis albopunctata* | C 565 | Estação Ecológica de Itirapina, SP, Brazil | JQ268476 | KC180238 | KC180138 | KC179848 |
| *Chiasmocleis albopunctata* | C572 | Estação Ecológica de Itirapina, SP, Brazil | KC180023 | KC180239 | KC180191 | KC179849 |
| *Chiasmocleis alagoanus* | C 2683 | Mata do Catolé, Maceió, Alagoas. Brazil | KC180030 | KC180237 | KC180159 | KC179846 |
| *Chiasmocleis alagoanus* | C 2682 | Mata do Catolé, Maceió, Alagoas, Brazil | KC180026 | KC180236 | KC180160 | KC179845 |
| *Chiasmocleis schubarti* | CFBH 9332 | R.B. de Sooretama, Linhares, ES, Brazil | None | KC180247 | KC180192 | KC179859 |
| *Chiasmocleis schubarti* | CFBH 9331 | R.B. de Sooretama, Linhares, ES, Brazil | KC180071 | KC180246 | KC180122 | KC179858 |
| *Chiasmocleis carvalhoi* | C 73 | Picinguaba, Ubatuba, SP, Brazil | KC180040 | KC180241 | KC180202 | KC179853 |
| *Chiasmocleis carvalhoi* | C 76 | Picinguaba, Ubatuba, SP, Brazil | KC180063 | KC180242 | KC180163 | KC179854 |
| *Chiasmocleis capixaba* | C 1437 | Linhares, ES, Brazil | KC180044 | KC180234 | KC180193 | KC179851 |
| *Chiasmocleis capixaba* | C 1438 | Linhares, ES, Brazil | KC179977 | KC180235 | KC180168 | KC179852 |
| *Dasypops schirchi* | T6 | Reserva do Vale, Linhares, ES, Brazil | KC180048 | KC180250 | KC180156 | KC179869 |
| *Myersiella microps* | M2358 | Pilar do Sul, SP, Brazil | KC180068 | KC180278 | KC180182 | KC179920 |
| *Myersiella microps* | M1131 | No locality data | KC180038 | KC180277 | KC180201 | KC179919 |
| *Myersiella microps* | Zaher | Serra Do Mar, Sao Paulo, Brazil | KC179973 | KC180279 | KC180210 | KC179921 |
| *Stereocyclops incrassatus* | MUFAI 2482 | Barra Nova, Marechal Deodoro, Alagoas, Brazil | KC180046 | None | KC180195 | KC179950 |
| *Stereocyclops incrassatus* | MUFAI 2483 | Barra Nova, Marechal Deodoro, Alagoas, Brazil | KC180045 | None | KC180165 | None |
| *Hyophryne histrio* | MNRJ 38931 | Bahia, Brazil | KC180064 | None | KC180115 | KC179890 |
| *Stereocyclops* sp. | A 399 | Itaúnas, Conceição da Barra, ES, Brazil | KC180014 | KC180231 | KC180178 | None |
| *Stereocyclops incrassatus* | CFBH 9334 | Fazenda Deus Dará, Linhares, ES, Brazil | None | None | KC180079 | KC179948 |
| *Stereocyclops incrassatus* | CFBH 9335 | Fazenda Deus Dará, Linhares, ES, Brazil | None | None | KC180105 | KC179949 |
| *Arcovomer passarellii* | A 1452 | Vitoria, ES, Brazil | KC180021 | None | KC180214 | KC179836 |
| *Arcovomer passarellii* | A 466 | Picinguaba, Ubatuba, SP, Brazil | KC180077 | None | KC180153 | KC179837 |
| *Altigius alios* | KU 215544 | Cuzco Amazonico, Madre de Dios, Peru | KC179975/KC180022 | KC180273 | KC180129/94 | KC179833/34 |
| *Hamptophryne boliviana* | JMP 216 | Bolivia | KC179988 | None | KC180176 | KC179886 |
| *Hamptophryne boliviana* | WED 57567 | Cuzco Amazonico, Madre de Dios, Peru | KC179987 | KC180316 | KC180154 | KC179887 |
| *Hamptophryne boliviana* | WED 57569 | Cuzco Amazonico, Madre de Dios, Peru | KC179976 | KC180317 | KC180181 | KC179888 |
| *Hamptophryne boliviana* | 44 2003 | Nacebe, Pando, Bolivia | KC180036 | None | KC180150 | None |
| *Dermatonotus muelleri* | D1344 | Pontalina, GO, Brazil | KC180037 | KC180249 | KC180144 | KC179871 |
| *Dermatonotus muelleri* | T7 | Paraguay | KC179984 | None | KC180157 | KC179870 |
| *Dermatonotus muelleri* | JMP | Bolivia | KC179985 | KC180251 | KC180189 | None |
| *Chiasmocleis panamensis* | AJC 988 | Peninsula Gigante, Lake Gatún, Panamá, Panama | KC179980 | KC180232 | KC180141 | KC179857 |
| *Elachistocleis ovalis* | NKAG 6488 | No locality data | KC180001 | None | KC180111 | KC179877 |
| *Elachistocleis bicolor* | ZVCB 10599 | Valle Edén, Tacuarembó, Uruguay | KC180005 | KC180318 | KC180164 | KC179874 |
| *Elachistocleis ovalis* | E54 | Lago Maracaibo, Santa Barbara de Zulia, Zulia, Venezuela | KC179999 | KC180252 | KC180135 | KC179879 |
| *Elachistocleis ovalis* | E53 | No locality data | KC180069 | KC180254 | KC180124 | KC179876 |
| *Elachistocleis ovalis* | E71 | Acequias Barina, Venezuela | None | KC180255 | KC180126 | KC179880 |
| *Elachistocleis bicolor* | E47 2003 | Nacebe, Pando, Bolivia | KC179982 | None | KC180080 | KC179875 |
| *Elachistocleis ovalis* | USFS 8793 | Brazil | JQ268479 | KC180312 | KC180207 | KC179878 |
| *Elachistocleis surinamensis* | E51 | Trincheras, rio Caura, Bolivar, Venezuela | KC180031 | KC180253 | KC180120 | KC179873 |
| *Relictivomer pearsei* | JDL 26585 43 | Colombia | KC179979 | KC180226 | KC180179 | KC179941 |
| *Relictivomer pearsei* | JDL 26194 45 | Colombia | KC179983 | KC180227 | KC180152 | KC179942 |
| *Gastrophryne elegans* | RdS 726 | Cockscomb Basin Wildlife Sanctuary, Stann Creek District, Belize | DQ283426 | DQ283019 | KC180151 | DQ283735 |
| *Gastrophryne carolinensis* | RdS 363 | Coldspring, San Jacinto Co., TX, USA | JQ268466 | KC180291 | KC180166 | None |
| *Gastrophryne olivacea* | RdS 295 | Stangel Ranch, Austin, Texas, USA | JQ268448 | KC180290 | KC180180 | KC179881 |
| *Hypopachus ustum* | UTA A-61716 (JAC 24021) | Mexico: Michoacan: HWY 200 | JQ268461 | KC180263 | KC180125 | KC179882 |
| *Hypopachus pictiventris* | 8850 | Costa Rica | JQ268462 | KC180259 | KC180118 | KC179885 |
| *Hypopachus barberi* | KU 291248 | Cerro El Pital, Chalatenango, El Salvador | KC179986 | KC180276 | KC180206 | KC179892 |
| *Hypopachus barberi* | ENS 8580A | Carretera Salomá-Pantin, Baja Verapaz, Guatemala | JQ268444 | KC180257 | KC180197 | KC179893 |
| *Hypopachus barberi* | UTA A-50963 (ENS 8049) | Uspantan, Quiche, Guatemala | JQ268445 | KC180256 | KC180212 | KC179897 |
| *Hypopachus barberi* | UTA A-55222 (JAC 19613) | Uspantan, Quiche, Guatemala | JQ268453 | KC180261 | KC180211 | KC179894 |
| *Hypopachus variolosus* | UTA A-53757 (MSM722) | Santa Rosa National Park, Liberia, Guanacaste, Costa Rica | None | None | KC180132 | KC179901 |
| *Hypopachus variolosus* | JHM 666 | Reserva Biologi Carara, Puntarenas, Costa Rica | KC180061 | KC180265 | KC180140 | KC179900 |
| *Hypopachus variolosus* | UTA A-51790 (ENS9818) | Nicaragua: Matagalpa: Comerca Penas Blancas | JQ268428 | KC180258 | KC180198 | KC179898 |
| *Hypopachus variolosus* | UTA A-60454 (JAC 24359) | Mexico: Campeche: Xpujil: Bel-Ha | JQ268475 | KC180264 | KC180146 | KC179899 |
| *Hypopachus variolosus* | UTA A-50968 (MSM 93) | Guatemala: Baja Verapaz: Salama | KC179978 | KC180281 | KC180196 | KC179895 |
| *Hypopachus variolosus* | UTA A-57702 (JAC 23325) | Carretera Sta. Maria de los Angeles-Bolanos Rio Cartagena, Jalisco, Mexico | JQ268474 | KC180262 | KC180185 | KC179896 |
