# Supplementary material for: Molecular phylogeny of microhylid frogs (Anura: Microhylidae) with emphasis on relationships among New World genera
Source: BMC Evol Biol. 2012 Dec 10;12:241. doi: 10.1186/1471-2148-12-241 (PMC3561245; doi:10.1186/1471-2148-12-241)
Supplement: Additional file 2 — Appendix 2. [file 1471-2148-12-241-S2.doc]

| **GenBank Samples** | **16S** | **TYR** | **BDNF** | **28S** |
| --- | --- | --- | --- | --- |
| *Scaphiopus* | DQ283150 | AB612073 | AB612074 | DQ283547 |
| *Alytes* | DQ283112 | EF407506 | EF407511 | DQ283510 |
| *Xenopus laevis* | Y10943 | AY341764 | JQ626687 | X59734 |
| *Hemisus marmoratus* | None | EF395975 | EF396011 | None |
| *Breviceps fuscus* | None | EF395962 | EF395997 | None |
| *Breviceps mossambicus* | None | EF395963 | EF395998 | DQ283546 |
| *Callulina kreffti* | None | DQ347189 | EF396000 |  |
| *Hoplophryne rogersi* | DQ283419 | EF395976 | EF396012 | DQ283730 |
| *Scaphiophryne calcarata* | None | EF395991 | EF396032 | None |
| *Scaphiophryne madagascariensis* | None | AB612003 | AB612004 | None |
| *Scaphiophryne marmorata* | AY843751 | AY844175 | None | DQ283720 |
| *Discophus antongilii* | None | EF395970 | EF396005 | None |
| *Discophus guineti* | DQ283434 | AB611891 | AB611892 | DQ283743 |
| *Discophus insularis* | None | EF395971 | EF396006 | None |
| *Aphantophryne pansa* | DQ283195 | None | None | DQ283578 |
| *Choerophryne sp.* | DQ283207 | None | None | DQ283583 |
| *Cophixalus sp.* 1 | DQ347050 | DQ347183 | None | None |
| *Cophixalus sp.* 2 | None | None | EF396003 | None |
| *Cophixalus sphagnicola* | DQ283206 | None | None | DQ283582 |
| *Copiula sp* | DQ283208 | None | None | DQ283584 |
| *Gastrophrynoides immaculatus* | AB611908 | AB611903 | AB611904 | None |
| *Genyophryne thomsoni* | DQ283209 | None | None | DQ283585 |
| *Liophryne rhododactyla* | DQ283199 | None | None | DQ283580 |
| *Oreophryne brachypus* | DQ283194 | None | None | DQ283577 |
| *Phrynomantis bifasciatus* | DQ283154 | EF395984 | EF396026 | DQ283545 |
| *Phrynomantis microps* | None | AB611979 | None | None |
| *Kalophrynus interlineatus* | None | AB611911 | AB611912 | None |
| *Kalophrynus pleurostigma* | DQ283146 | AB611919 | AB611920 | DQ283537 |
| *Otophryne pyburni* | None | EF395982 | EF396023 | None |
| *Calluella guttulata* 1 | DQ283144 | EF395964 | EF395999 | DQ283536 |
| *Calluella guttulata* 2 | EF017956 | AB611863 | AB611864 | None |
| *Chaperina fusca* | DQ283145 | AB611867 | AB611868 | None |
| *Glyphoglossus molossus* | None | EF395974 | EF396009 | None |
| *Kaluola pulchra* 1 | DQ283398 | EF395978 | EF396015 | DQ283727 |
| *Kaluola pulchra* 2 | DQ283397 | None | None | DQ283711 |
| *Kaluola taprobanica* 1 | None | AB611924 | AB611925 | None |
| *Kaluola taprobanica* 2 | None | AF249163 | None | None |
| *Metaphrynella pollicaris* | AB634692 | AB611929 | AB611930 | None |
| *Metaphrynella sundada* | EF017954 | AB611937 | AB611938 | None |
| *Microhyla annectens* | None | None | AB611943 | None |
| *Microhyla heymonsi* | DQ283382 | EF395979 | EF396020 | DQ283697 |
| *Microhyla marmorata* | None | AB611950 | AB611951 | None |
| *Microhyla okinavensis* | None | AB611958 | AB611959 | None |
| *Microhylapulchra* | None | EF395980 | EF396021 | None |
| *Microhyla sp* | DQ283422 | AF249162 | None | DQ283732 |
| *Mycryletta inornata* | AB634695 | EF395981 | EF396022 | None |
| *Phrynella pulchra* | AB634694 | AB611971 | AB611972 | None |
| *Ramanella montana* | AB612000 | AB611995 | AB611996 | None |
| *Ramanella obscura* | AF215382 | EF395989 | EF396030 | None |
| *Anodonthyla boulengerii* | None | EF395959 | None | None |
| *Anodonthyla montana* | None | EF395960 | EF395995 | None |
| *Plethodontohyla brevipes* | None | EF395987 | EF396029 | None |
| *Plethodontohyla inguinalis* | None | AB611987 | AB611988 | None |
| *Plethodontohyla sp.* | DQ283409 | None | None | DQ283719 |
| *Platypelis grandis* | DQ283410 | EF395986 | EF396027 | DQ283721 |
| *Stumpffia psologlossa* | DQ283411 | EF395992 | EF396033 | None |
| *Stumpffia pygmaea* | None | EF395993 | EF396034 | None |
| *Chiasmocleis hudsoni* | None | EF395967 | EF396001 | None |
| *Chiasmocleis shudikarensis* | None | EF395966 | EF396002 | None |
| *Dasypops schirchi* | DQ283095 | None | None | DQ283497 |
| *Ctenophryne geayi* | None | AB611883 | AB611884 | DQ283698 |
| *Dermatonotus muelleri* | AY948747 | EF395969 | EF396004 | None |
| *Elachistocleis ovalis* | DQ283405 | EF395972 | None | None |
| *Gastrophryne elegans* | DQ283426 | None | None | None |
| *Hamptophryne boliviana* | DQ283438 | None | None | DQ283747 |
